# Association of an Advance Care Planning Video and Communication Intervention With Documentation of Advance Care Planning Among Older Adults: A Nonrandomized Controlled Trial

**DOI:** 10.1001/jamanetworkopen.2022.0354

**Published:** 2022-02-24

**Authors:** Angelo E. Volandes, Sophia N. Zupanc, Michael K. Paasche-Orlow, Joshua R. Lakin, Yuchiao Chang, Edith A. Burns, Nancy A. LaVine, Maria T. Carney, Diana Martins-Welch, Kaitlin Emmert, Jennifer E. Itty, Edward T. Moseley, Aretha D. Davis, Areej El-Jawahri, Daniel A. Gundersen, Gemmae M. Fix, Andrea M. Yacoub, Pamela Schwartz, Shira Gabry-Kalikow, Cynthia Garde, Jonathan Fischer, Lori Henault, Leah Burgess, Julie Goldman, Anne Kwok, Nimisha Singh, Armando L. Alvarez Suarez, Valeria Gromova, Sonia Jacome, James A. Tulsky, Charlotta Lindvall

**Affiliations:** 1 Harvard Medical School, Boston, Massachusetts; 2 Department of Medicine, Massachusetts General Hospital, Boston; 3 ACP Decisions, Boston, Massachusetts; 4 Department of Psychosocial Oncology and Palliative Care, Dana-Farber Cancer Institute, Boston, Massachusetts; 5 Department of Medicine, Boston University School of Medicine, Boston Medical Center, Boston, Massachusetts; 6 Department of Medicine, Brigham and Women’s Hospital, Boston, Massachusetts; 7 Center for Health Innovations and Outcomes Research, Feinstein Institutes for Medical Research, Manhasset, New York; 8 Department of Medicine, Zucker School of Medicine Hosftra/Northwell, New Hyde Park, New York; 9 Division of Population Sciences, Dana-Farber Cancer Institute, Boston, Massachusetts; 10 Center for Healthcare Organization and Implementation Research, Veterans Affairs Bedford Healthcare System, Bedford, Massachusetts; 11 Department of Community Health and Family Medicine, Hospice and Palliative Care, Duke University Health System, Durham, North Carolina

## Abstract

**Importance:**

COVID-19 has disproportionately killed older adults and racial and ethnic minority individuals, raising questions about the relevance of advance care planning (ACP) in this population. Video decision aids and communication skills training offer scalable delivery models.

**Objective:**

To assess whether ACP video decision aids and a clinician communication intervention improved the rate of ACP documentation during an evolving pandemic, with a focus on African American and Hispanic patients.

**Design, Setting, and Participants:**

The Advance Care Planning: Communicating With Outpatients for Vital Informed Decisions trial was a pre-post, open-cohort nonrandomized controlled trial that compared ACP documentation across the baseline pre–COVID-19 period (September 15, 2019, to March 14, 2020), the COVID-19 wave 1 period (March 15, 2020, to September 14, 2020), and an intervention period (December 15, 2020, to June 14, 2021) at a New York metropolitan area ambulatory network of 22 clinics. All patients 65 years or older who had at least 1 clinic or telehealth visit during any of the 3 study periods were included.

**Main Outcomes and Measures:**

The primary outcome was ACP documentation.

**Results:**

A total of 14 107 patients (mean [SD] age, 81.0 [8.4] years; 8856 [62.8%] female; and 2248 [15.9%] African American or Hispanic) interacted with clinicians during the pre–COVID-19 period; 12 806 (mean [SD] age, 81.2 [8.5] years; 8047 [62.8%] female; and 1992 [15.6%] African American or Hispanic), during wave 1; and 15 106 (mean [SD] 80.9 [8.3] years; 9543 [63.2%] female; and 2535 [16.8%] African American or Hispanic), during the intervention period. Clinicians documented ACP in 3587 patients (23.8%) during the intervention period compared with 2525 (17.9%) during the pre–COVID-19 period (rate difference [RD], 5.8%; 95% CI, 0.9%-7.9%; *P* = .01) and 1598 (12.5%) during wave 1 (RD, 11.3%; 95% CI, 6.3%-12.1%; *P* < .001). Advance care planning was documented in 447 African American patients (30.0%) during the intervention period compared with 233 (18.1%) during the pre–COVID-19 period (RD, 11.9%; 95% CI, 4.1%-15.9%; *P* < .001) and 130 (11.0%) during wave 1 (RD, 19.1%; 95% CI, 11.7%-21.2%; *P* < .001). Advance care planning was documented for 222 Hispanic patients (21.2%) during the intervention period compared with 127 (13.2%) during the pre–COVID-19 period (RD, 8.0%; 95% CI, 2.1%-10.9%; *P* = .004) and 82 (10.2%) during wave 1 (RD, 11.1%; 95% CI, 5.5%-14.5%; *P* < .001).

**Conclusions and Relevance:**

This intervention, implemented during the evolving COVID-19 pandemic, was associated with higher rates of ACP documentation, especially for African American and Hispanic patients.

**Trial Registration:**

ClinicalTrials.gov Identifier: NCT04660422

## Key Points

**Question:**

Can an advance care planning (ACP) video and communication intervention promote ACP for elderly patients during the ongoing COVID-19 pandemic?

**Findings:**

This pre-post, open-cohort nonrandomized controlled trial compared ACP documentation during three 6-month periods: pre–COVID-19 (14 107 patients), COVID-19 wave 1 (12 806 patients), and an intervention period (15 106 patients). The ACP documentation rates were 17.9% in the pre–COVID-19 period, 12.5% in the COVID-19 wave 1 period, and 23.7% in the intervention period; ACP rates during the intervention period were highest compared with the 2 other periods.

**Meaning:**

The use of an ACP video and communication intervention may promote ACP for elderly adults during the evolving COVID-19 pandemic.

## Teaser

This nonrandomized controlled trial assesses whether an advance care planning intervention improved rates of advance care planning in older adults during the COVID-19 pandemic.

## Introduction

The COVID-19 pandemic has resulted in more than 800 000 deaths in the US, 77% of which were among people 65 years or older and 33% among African American and Hispanic individuals.^1^ Many of these patients faced emergency decisions about the use of life-sustaining treatments, an eventuality for which they and their families were often unprepared.^2^ Advance care planning (ACP) seeks to help patients receive care that reflects what matters most to them.^3^ This process anchors on educating patients about the medical care they may receive and elucidating their informed preferences through iterative conversations and discussions.^3,4^ In addition, ACP often includes choosing and preparing another trusted person to make medical decisions in the event that an individual no longer can do so themselves.^3,4,5^

In normal times, such advance decision-making may seem hypothetical for many people who cannot easily imagine future health states.^6^ However, the COVID-19 pandemic has presented a unique ACP context, especially for elderly, African American, and Hispanic patients for whom the pandemic has made such choices a familiar reality.^7^ Advance care planning is no longer hypothetical. Patients have a high probability of facing discrete choices (eg, mechanical ventilation) that require in-the-moment decision-making. Furthermore, many clinicians working during the pandemic are intimately familiar with the dire situations that arise and hope to ease the burden of decision-making on patients and families.

In prior work,^8,9,10,11,12,13^ video decision aids and clinician communication skills training have each shown promise in promoting ACP. However, scaling for each of these interventions has proved challenging,^14,15^ and, overall, ACP interventions that have focused on patient engagement or clinician skills training alone have been suboptimal.^6,16^ The immediate, tangible threats faced by patients and the rapid expansion of technology use during the COVID-19 pandemic^17^ afforded an opportunity to combine these intervention types to test the scaling of an integrated ACP approach in a large, diverse population of outpatients aged 65 years or older. The Advance Care Planning: Communicating With Outpatients for Vital Informed Decisions (ACP-COVID) study sought to assess whether a rapid, large-scale implementation of an intervention that consisted of ACP video decision aids for patients and clinician communication skills training in the ambulatory care setting was associated with an increase in the rate of ACP documentation during an evolving pandemic. A secondary aim was to explore the intervention association with ACP documentation for African American and Hispanic patients.

## Methods

### Trial Design and Oversight

**Figure 1.**
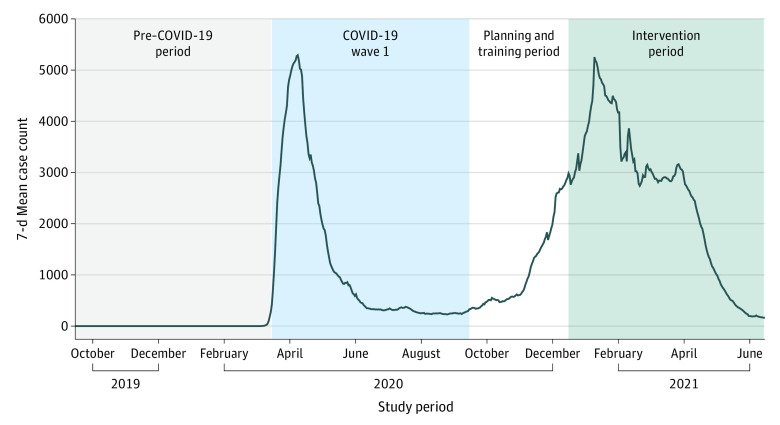
Study Timeline With Overlay of COVID-19 Cases in New York City

The ACP-COVID study was a pre-post, open-cohort nonrandomized controlled trial that aimed to evaluate an ACP intervention in older patients during an evolving pandemic. Three prespecified periods were chosen to compare ACP documentation: a baseline period before COVID-19 (pre–COVID-19; September 15, 2019, to March 14, 2020), the first wave of COVID-19 cases in New York City (wave 1; March 15, 2020, to September 14, 2020),^18^ and the intervention period (December 15, 2020, to June 14, 2021) (Figure 1). Our primary comparison was between the intervention period and the wave 1 period (see Supplement 1 for the trial protocol). The trial was approved by the Dana-Farber Cancer Institute Institutional Review Board, and informed consent was waived because this was deemed a minimal risk trial. This study followed the Transparent Reporting of Evaluations With Nonrandomized Designs (TREND) reporting guideline.

### Patients and Clinicians

All patients 65 years or older who had at least 1 in-person or telehealth visit to a participating clinic during any of the 3 periods were included in the study. Patients were recruited from 22 outpatient clinics of Northwell Health, the largest health care organization in New York. All clinicians affiliated with these clinics were invited to participate in the intervention. Training sessions were held during the planning and training period (Figure 1).

### Intervention

The ACP intervention consisted of video decision aids for patients and communication skills training for clinicians. Video decision aids included ACP-related videos and videos on COVID-19 and vaccinations because of the salience of these topics.^19^ The ACP Decisions video decision aids were disseminated to all patients 65 years or older 1 to 2 weeks before a clinic or telehealth appointment through links sent via text message, email, or mail, whichever means was available. The videos were also available to patients in person before or during their clinic visit. Patients chose which videos they wished to view from the following options: Choosing a Health Care Proxy, Having an ACP Conversation, What Is COVID-19, and COVID-19 Vaccinations (see the eMethods in Supplement 2 for video descriptions). We included COVID-19 videos for adults, teens, and children to engage patients and their families.^19^ All videos were designed for a health literacy level of less than sixth grade, and video images of patients and clinicians were diverse, reflecting the community being served.^20,21^ Videos were available in English and Spanish. Clinic-level data on video use were monitored weekly, and dissemination efforts regarding modality (ie, texting, email, or mailings) were modulated weekly based on video use data.

Clinician communication skills training was offered to all clinicians affiliated with the practices. Clinicians participated in a remote, 4-hour, VitalTalk-designed interactive training via Zoom in which they practiced introducing the concept of ACP, discussing prognosis, exploring cultural concerns unique to minority communities, and addressing common COVID-19 ACP scenarios.^22,23,24^ VitalTalk facilitators used a standardized teaching method that included clinician observation of exemplar communication behaviors, practice with live actor patients, and immediate focused feedback.

### Outcomes and Assessment

Our primary outcome was the documentation of ACP in a clinician’s electronic health record (EHR) note. This documentation included any notation of a discussion about goals of care and preferences for future medical care, palliative care, hospice, or a health care proxy, each of which was evaluated independently as well. These preferences were identified using human-assisted natural language processing (NLP) as described in earlier studies.^25,26,27,28,29,30^ Prior work^25,31^ found that an NLP search of clinician notes was more accurate in finding these outcomes than extracting structured data elements (eg, uploaded ACP documents) from the EHR (additional details about the study NLP methods are provided in eTable 1 in Supplement 2). Our secondary outcome was ACP rates in prespecified racial and ethnic subgroups.

### Statistical Analysis

The primary analysis included all eligible patients in the 3 prespecified periods. We compared the outcome measures mentioned above between the intervention and the 2 control periods, adjusting for patient characteristics (age, sex, race and ethnicity, marital status, location, and number of encounters during each 6-month period). The primary comparison was between the intervention and wave 1 periods. Rate differences (RDs) were estimated using Poisson models with identity link functions.^32^ We used the generalized estimating equations approach to account for patients within clinic practice clustering and the repeated measures over time from the same individuals. As a sensitivity analysis, we limited the cohort to those who appeared in all 3 comparison periods. We conducted prespecified subgroup analyses (with individuals of any non-White racial minority and Hispanic ethnicity combined and with individuals of each racial and ethnic minority separately). A 2-sided *P* < .05 was considered to be statistically significant.

We conservatively estimated that approximately 7800 patients from 150 clinicians would be eligible for the study at each period and 85% of patients would overlap from one period to the next. With 3 periods, we anticipated including a total of 10 139 unique patients in the study. In the most conservative scenario, with each clinician contributing a mean of 44 patients, the design effect was estimated as 3.2, assuming an intracluster correlation of 0.05, which corresponds to an effective sample size of 2098 appearing in both periods. Preliminary estimates indicated the rate for ACP documentation (primary outcome) would be approximately 10% in the pre–COVID-19 period and 20% during wave 1. The study was designed with more than 95% power to detect a 5% absolute increase in outcome, with a 2-sided *P* < .05 considered to be statistically significant.

For the subgroup analysis, preliminary estimates suggested that 30% of the population would be non-White. When limited to the non-White subgroups, we expected 2340 patients in each of the 2 periods. In the most conservative scenario, with each clinician contributing a mean of 13 patients, the design effect was estimated to be 1.6, assuming an intracluster correlation of 0.05, which corresponds to an effective sample size of 1233 appearing in both periods. The study would have more than 95% power to detect a 5% absolute increase in outcome, with a 2-sided *P* < .05 considered to be statistically significant. All statistical analyses were conducted using SAS software, version 9.4 (SAS Institute Inc).

## Results

### Patients and Clinicians

A total of 14 107 patients (mean [SD] age, 81.0 [8.4] years; 8856 [62.8%] female; and 2248 [15.9%] African American or Hispanic) interacted with clinicians during the pre–COVID-19 period, 12 806 (mean [SD] age, 81.2 [8.5] years; 8047 [62.8%] female; and 1992 [15.6%] African American or Hispanic) during wave 1, and 15 106 (mean [SD] 80.9 [8.3] years; 9543 [63.2%] female; and 2535 [16.8%] African American or Hispanic) during the intervention period. Patient characteristics were similar across the 3 study periods (Table 1). Race or ethnicity data were missing for 891 patients (6.3%) in the pre–COVID-19 period, 883 patients (6.9%) in the wave 1 period, and 1143 patients (7.6%) in the intervention period. Of the 219 clinicians affiliated with the participating outpatient clinics, 185 (84.5%) underwent the intervention communication training.

**Table 1.** Characteristics of the Study Population^a^

Characteristic	Pre–COVID-19 (n = 14 107)	COVID-19 wave 1 (n = 12 806)	Intervention period (n = 15 106)
Age, mean (SD), y	81.0 (8.4)	81.2 (8.5)	80.9 (8.3)
Sex			
Female	8856 (62.8)	8047 (62.8)	9543 (63.2)
Male	5251 (37.2)	4759 (37.2)	5563 (36.8)
Race and ethnicity			
Hispanic	961 (6.8)	806 (6.3)	1046 (6.9)
Non-Hispanic			
Asian	665 (4.7)	568 (4.4)	742 (4.9)
Black	1287 (9.1)	1186 (9.3)	1489 (9.9)
White	9600 (68.1)	8717 (68.1)	9904 (65.6)
Other^b^	703 (5.0)	646 (5.0)	782 (5.2)
Unknown	891 (6.3)	883 (6.9)	1143 (7.6)
Marital status			
Married	7996 (56.7)	7124 (55.6)	8370 (55.4)
Widowed	2232 (15.8)	2097 (16.4)	2236 (14.8)
Divorced or separated	1014 (7.2)	944 (7.4)	1103 (7.3)
Single	1815 (12.9)	1610 (12.6)	1973 (13.1)
Other	68 (0.5)	60 (0.5)	74 (0.5)
Unknown	982 (7.0)	971 (7.6)	1350 (8.9)
Clinic visits			
1	5984 (42.2)	5655 (44.2)	6112 (40.5)
2	3047 (21.6)	2528 (19.7)	3239 (21.4)
3-4	2340 (16.6)	2122 (16.6)	2714 (18.0)
≥5	2736 (19.4)	2501 (19.5)	3041 (20.1)
Patients with telehealth encounters	523 (3.7)	3228 (25.2)	2262 (15.0)

^a^ Data are presented as number (percentage) of patients unless otherwise indicated.

^b^ The other category encompasses all individuals who were not Native Hawaiian, Hispanic, non-Hispanic White, non-Hispanic African American, or non-Hispanic Asian or those who had missing, declined/not reported, or unknown for their race and ethnicity data.

### Video Decision Aid Use

**Figure 2.**
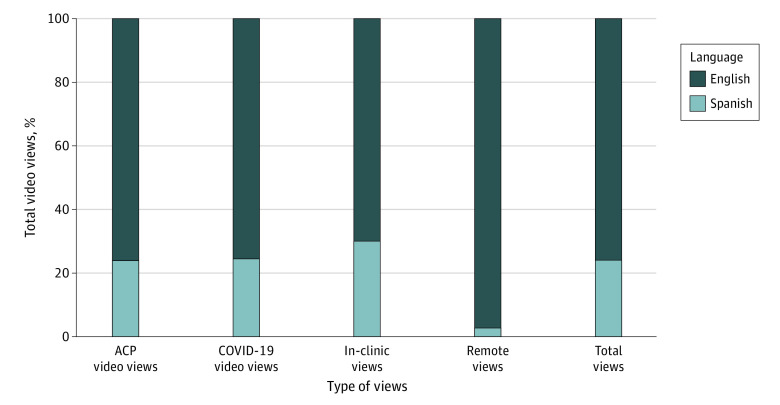
Video Views Stratified by Language ACP indicates advance care planning.

A total of 5302 videos were viewed during the intervention period, including 4023 (75.9%) in English and 1279 (24.1%) in Spanish. A total of 4163 videos (78.5%) were viewed in person during a clinic visit, and 1139 (21.5%) were viewed via a web link. Of the 5302 videos viewed, 3587 (67.7%) contained ACP-related content, whereas 1715 (32.3%) were related to COVID-19. Of the 5302 videos viewed, 4752 (89.6%) had a watch percentage of 50% or greater, and 550 (10.4%) had a watch percentage of less than 50% (Figure 2; eTables 2 and 3 in Supplement 2).

### Primary Outcome

**Figure 3.**
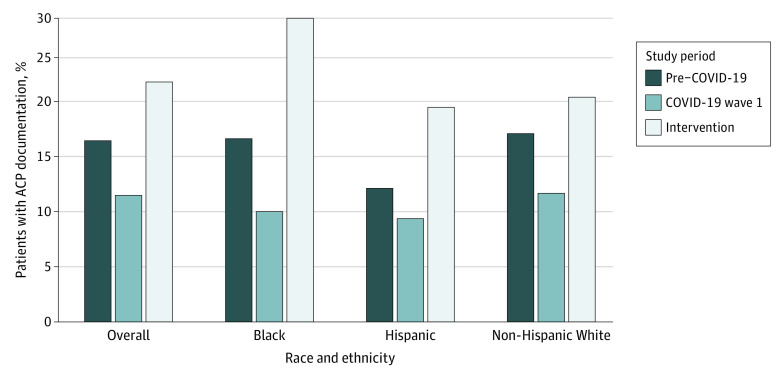
Rates of Advance Care Planning (ACP) Documentation Overall and Among Minority Subgroups in the 3 Study Periods

Advance care planning documentation was identified in 3587 patients (23.8%) during the intervention period compared with 2525 patients (17.9%) during the pre–COVID-19 period (RD, 5.8%; 95% CI, 0.9%-7.9%; *P* = .01) and 1598 (12.5%) during wave 1 (RD, 11.3%; 95% CI, 6.3%-12.1%; *P* < .001) (Figure 3). The results were similar in the sensitivity analysis limited to the 7180 patients who appeared in all 3 comparison periods (18.5% in the pre–COVID-19 period, 11.4% in wave 1, and 21.8% in the intervention period) (eTables 4 and 5 in Supplement 2).

### Secondary Outcomes

All 5 elements of ACP documentation (discussions about goals of care and preferences for medical care, palliative care, hospice, and health care proxy) were greatest during the intervention period (Table 2). Goals of care were identified for 3506 patients (23.2%) during the intervention period compared with 2383 patients (16.9%) during the pre–COVID-19 period (RD, 6.3%; 95% CI, 1.0%-8.0%; *P* = .01) and 1512 (11.8%) during wave 1 (RD, 11.4%; 95% CI, 6.0%-11.9%; *P* < .001). A health care proxy was identified for 2670 patients (17.7%) during the intervention period compared with 1637 patients (11.6%) during the pre–COVID-19 period (RD, 6.1%; 95% CI, 1.5%-8.0%; *P* = .004) and 1024 (8.0%) during wave 1 (RD, 9.7%; 95% CI, 5.5%-11.2%; *P* < .001).

**Table 2.** Rate Differences Between Intervention and Baseline Periods Using Generalized Linear Regression Models With Generalized Estimating Equations

Outcome	Intervention (n = 15 106), No. (%)	Pre–COVID-19 (n = 14 107)			COVID-19 wave 1 (n = 12 806)		
		No. (%)	RD (95% CI)^a^	*P* value^a^	No. (%)	RD (95% CI)^a^	*P* value^a^
**Primary outcome**							
ACP documentation							
Overall	3587 (23.8)	2525 (17.9)	5.8 (0.9 to 7.9)	.01	1598 (12.5)	11.3 (6.3 to 12.1)	<.001
Subgroups							
Non-Hispanic White	2192 (22.1)	1791 (18.7)	3.5 (−1.4 to 6.4)	.21	1105 (12.7)	9.5 (4.9 to 11.1)	<.001
Unknown	336 (29.4)	136 (15.3)	NA	NA	145 (16.4)	NA	NA
Minority^b^	1059 (26.1)	598 (16.5)	9.6 (3.5 to 11.7)	<.001	348 (10.9)	15.2 (9.2 to 16.7)	<.001
Hispanic	222 (21.2)	127 (13.2)	8.0 (2.1 to 10.9)	.004	82 (10.2)	11.1 (5.5 to 14.5)	<.001
Non-Hispanic Asian	200 (27.0)	118 (17.7)	9.2 (3.8 to 13.1)	<.001	60 (10.6)	16.4 (10.6 to 19.7)	<.001
Non-Hispanic Black	447 (30.0)	233 (18.1)	11.9 (4.1 to 15.9)	<.001	130 (11.0)	19.1 (11.7 to 21.2)	<.001
**Secondary outcomes**							
Goals of care	3506 (23.2)	2383 (16.9)	6.3 (1.0 to 8.0)	.01	1512 (11.8)	11.4 (6.0 to 11.9)	<.001
Palliative care	61 (0.4)	29 (0.2)	0.2 (0.0 to 0.4)	.03	20 (0.2)	0.2 (0.0 to 0.4)	.02
Hospice	164 (1.1)	96 (0.7)	0.4 (−0.2 to 1.0)	.16	74 (0.6)	0.5 (0.2 to 0.9)	.001
Limitations on life-sustaining treatment	464 (3.1)	335 (2.4)	0.7 (−0.3 to 1.4)	.21	212 (1.7)	1.4 (0.8 to 2.2)	<.001
Surrogate decision maker	2670 (17.7)	1637 (11.6)	6.1 (1.5 to 8.0)	.004	1024 (8.0)	9.7 (5.5 to 11.2)	<.001

Abbreviations: ACP, advance care planning; NA, not applicable; RD, rate difference.

^a^ Model adjusted for age, sex, marital status, location, number of encounters, and race and ethnicity.

^b^ All individuals who did not have a race or ethnicity value of non-Hispanic White.

### Subgroup Analysis

The presence of ACP documentation among all patients of a racial minority was 26.1% (n = 1059) during the intervention period compared with 16.5% (n = 598) during the pre–COVID-19 period (RD, 9.6%; 95% CI, 3.5%-11.7%]; *P* < .001) and 10.9% (n = 348) during wave 1 (RD, 15.2%; 95% CI, 9.2%-16.7%; *P* < .001). The presence of ACP documentation among African American patients was 30.0% (n = 447) during the intervention period compared with 18.1% (n = 233) during the pre–COVID-19 period (RD, 11.9%; 95% CI, 4.1%-15.9%; *P* < .001) and 11.0% (n = 130) during wave 1 (RD, 19.1%; 95% CI, 11.7%-21.2%; *P* < .001). The presence of ACP documentation among Hispanic patients was 21.2% (n = 222) during the intervention period compared with 13.2% (n = 127) during the pre–COVID-19 period (RD, 8.0%; 95% CI, 2.1%-10.9%; *P* = .004) and 10.2% (n = 82) during wave 1 (RD, 11.1%; 95% CI, 5.5%-14.5%; *P* < .001) (Table 2 and Figure 3).

## Discussion

In this large, pragmatic, ambulatory care intervention in a diverse population affected by the COVID-19 pandemic, patients in the intervention period had an opportunity to watch video ACP decision aids and interact with clinicians trained in tailored communication skills. Video use was robust, and penetration of communication skills training was high, demonstrating successful intervention implementation. Documentation of ACP discussion was much greater during the intervention period compared with the pre–COVID-19 or COVID-19 wave 1 periods. The intervention was also associated with increased ACP documentation for African Americans and Hispanic patients.

Advance care planning interventions have had mixed results in prior trials,^6,16,33^ and the study of ACP during an evolving pandemic is unprecedented. The COVID-19 pandemic increased the relevance of ACP because patients were asked to consider potential scenarios that were familiar, highly relevant, and emotionally charged. The intervention period was associated with increased goals-of-care and health care proxy discussions, as the relevance and importance of ACP likely increased during the unfolding pandemic. Advance care planning may be more urgent now than ever before because of the COVID-19 pandemic.

The unequal impact of COVID-19 may explain why communities disproportionately affected by the pandemic had higher rates of ACP documentation. Specifically, African American and Hispanic patients were more likely to have ACP documentation during the intervention period compared with non-Hispanic White patients. Intervention benefit for African American and Hispanic patients was a goal of the project as reflected in the design of the videos and clinician training. However, it is possible that this effect also was due to higher exposure to serious COVID-19 illness in the families of African American and Hispanic patients.

Prior clinician incentives intended to promote ACP, such as Medicare ACP billing codes, have been suboptimal in encouraging ACP.^34^ Clinicians’ first-hand experience caring for patients with COVID-19 and graphic media images of overflowing intensive care units and mobile morgue units may have changed clinician attitudes to ACP. The 2022 ACP Healthcare Effectiveness Data and Information Set measure^35^ may also further boost ACP. The pandemic has raised the relevance of ACP for patients and clinicians, especially for people with direct exposure to death and dying, and has presented an opportunity to encourage more widespread use of ACP, an elusive goal for most health care systems during the last 3 decades.

Previous trials of ACP interventions have often fallen short because of suboptimal implementation.^14^ In this study, we were able to monitor fidelity to the intervention in real time. Video viewings were monitored weekly, and implementation strategies shifted to meet patients’ needs (eg, texting, in-person viewings, and mailings). Patients’ increased use of telehealth services and smartphone technology^17^ played an important role in exposure to the videos. Remote clinician training allowed for faster dissemination of the program and yielded nearly complete participation of clinicians in the training. We capitalized on expanded technology access for patients and the possibility of remote interactive trainings for clinicians; future interventions may benefit from such opportunities as well. Analysis is ongoing looking at the association of this intervention on COVID-19 vaccination rates and goal-concordant care.

### Limitations

Our findings must be considered in the context of several limitations. First, the intervention took place in an outpatient setting in a region with one of the worst death rates during the pandemic. Studying the intervention in other geographic locations and in the inpatient and emergency department settings would be of great interest.

Second, we looked at ACP documentation rates in the EHR during a 6-month intervention period. Long-term studies looking at care delivery and concordance with patient goals are ongoing, but because these may take years to complete, the knowledge generated will not be available to direct care during a developing pandemic. Assessment of ACP documentation allowed for rapid results with immediate application to the delivery of medical care.

Third, missing data present another limitation. Specifically, in our 3 study periods, race or ethnicity data were missing for 6.3% of patients in the pre–COVID-19 period, 6.9% in the wave 1 period, and 7.6% in the intervention period. Although these rates are within recommended guidelines for identifying hospitals with potentially unreliable race and ethnicity data, this level of missingness decreases the precision of our analyses.^36^

Fourth, potential unmeasured confounders may influence this trial because it was not randomized. For example, patients with worse health status are more likely to be approached by clinicians for ACP. Similarly, patients and clinicians exposed to death in the context of the pandemic may have an augmented interest in ACP.

Fifth, we were not able to track video use at the patient level in a manner that allowed direct linkage to EHR-based outcomes. Future work should follow patient viewing and direct causal linkage of video viewing with the outcomes of interest.

Sixth, our system for generating invitations for remote viewing was initially implemented in English. We expanded to Spanish late in the trial period. This delayed introduction certainly contributed to low remote video use among Spanish speakers.

Seventh, any pragmatic trial is subject to secular trends in potentially relevant phenomena, which is certainly the case during an evolving pandemic. Our prespecified intervention period corresponded with a significant second wave in New York City (Figure 1), which may have influenced the outcome.

## Conclusions

As one of the first large, descriptive, pragmatic ACP trials to be conducted during the COVID-19 pandemic, the ACP-COVID trial demonstrated a significant and clinically meaningful association of ACP documentation rates in a rapid and scalable manner that could be quickly implemented nationally. In addition, the intervention association was greater among African American and Hispanic patients compared with non-Hispanic White patients. Implications should be considered from the perspectives of numerous key stakeholders. For patients, caregivers, and clinicians, rapidly changing information needs to be disseminated in a meaningful way and in a fashion that honors and respects the diverse communities affected. The ACP-COVID intervention leveraged diverse video decision aids and remote clinician training to empower patients and clinicians by addressing urgent informational and emotional needs. For health care systems, corporate leaders, and government officials, the ACP-COVID intervention represents 1 of the first rapidly adoptable programs with significant association in promoting ACP, a widely used quality metric. Finally, for research studies, the use of patient video decision aids, clinician training, and NLP-assessed outcomes offers a powerful way forward to conduct similar large-scale trials in ACP and beyond.

## Notes

Supplement 1.
Trial Protocol

Supplement 2. **eMethods.** Study Organization and Committees **eTable 1.** Keyword Library Used for NLP Annotations **eTable 2.** Total Number of Video Views by Video Type, Language, and Modality **eTable 3.** Video View Watched Percentage **eTable 4.** Patient Characteristics Limited to Patients Who Appeared in All Three Periods (Sensitivity Analysis) **eTable 5.** NLP ACP Documentation Rates by Domain and Overall Limited to Patients Who Appeared in All Three Periods (Sensitivity Analysis) **eTable 6.** Rate Difference Between Intervention and Baseline Periods Using Generalized Linear Regression Models with Generalized Estimating Equations Limited to Patients Who Appeared in All Three Periods (Sensitivity Analysis)
eReferences

Supplement 3.
Data Sharing Statement
