# Supplementary material for: Association of an Advance Care Planning Video and Communication Intervention With Documentation of Advance Care Planning Among Older Adults: A Nonrandomized Controlled Trial
Source: JAMA Netw Open. 2022 Feb 24;5(2):e220354. doi: 10.1001/jamanetworkopen.2022.0354 (PMC8874350; doi:10.1001/jamanetworkopen.2022.0354)
Supplement: Supplement 1. — Trial Protocol [file jamanetwopen-e220354-s001.pdf]

PROTOCOL TITLE:

Advance Care Planning: Communicating with Outpatients for Vital Informed Decisions (ACP-COVID)

PRINCIPAL INVESTIGATORS:

James A. Tulsky

Dana Farber Cancer Institute

Angelo E. Volandes

Massachusetts General Hospital

VERSION NUMBER: 5

DATE: 5/4/2021

Table of Contents

1.0 Objectives 3
2.0 Background 4
3.0 Inclusion and Exclusion Criteria 6
4.0 Study-Wide Number of Subjects* 7
5.0 Study-Wide Recruitment Methods* 7
6.0 Multi-Site Research* 7
7.0 Study Timelines 9
8.0 Study Endpoints 10
9.0 Procedures Involved 10
10.0 Data and Specimen Banking 13
11.0 Data Management and Confidentiality 13
12.0 Provisions to Monitor the Data to Ensure the Safety of Subjects* 15
13.0 Withdrawal of Subjects 15
14.0 Risks to Subjects 15
15.0 Potential Benefits to Subjects 16
16.0 Sharing of Results with Subjects 17
17.0 Setting 17
18.0 Prior Approvals 18
19.0 Recruitment Methods 18
20.0 Local Number of Subjects 18
21.0 Provisions to Protect the Privacy Interests of Subjects 18
22.0 Consent Process 19

1.0 Objectives

The overall objective of this application is to reduce the burden of COVID-19 and its consequences for an aging U.S. population that may prefer to forgo aggressive interventions that are unlikely to help, and to die with loved ones present outside of the hospital setting. To accomplish this, we propose to conduct a Pre-Post trial using an open cohort design of a comprehensive telehealth advance care planning (ACP) Program among older patients in New York, one of the nation's COVID-19 epicenters. To address this gap, we have developed a **Comprehensive Telehealth ACP Program for COVID-19** that implements ACP routinely into primary medical care. The Comprehensive ACP Program combines two widely disseminated interventions to assess their impact when used concurrently. One is VitalTalk clinician communication skills training (www.vitaltalk.org) during which the primary care clinicians will practice ACP with simulated patient actors under the guidance of a trained VitalTalk facilitator. The second is the ACP Decisions video decision aids (www.acpdecisions.org) which are directed to patients and provide education about ACP. This inclusive ACP approach treats patients and clinicians as equal stakeholders providing both with the communication skills and tools needed to make decisions about COVID-19 medical care before the toughest choices arise.

The training is four cumulative hours, most of which is spent in communication skills work, and the remainder learning how to introduce the videos into one's practice. No patients participate in the training. The ambulatory practices included in this intervention gain access to the ACP Decisions videos, which can be introduced to patients. Videos may be seen in clinic or sent to patients' homes via an electronic or paper code. We have shown the efficacy of the elements of the Comprehensive ACP intervention in several randomized controlled trials (RCTs) for older patients.(1-5)

We will train 150 primary care clinicians caring for 13,000 diverse patients over the age of 65 from Northwell Health, a health care system in which we are already embedded with our ongoing grant. We will use Natural Language Processing (NLP) to abstract patient outcomes from the Electronic Health Record (EHR). *We hypothesize that a telehealth ACP Program will improve and sustain rates of ACP from the time that the intervention is implemented (11/01/2020 through 04/30/2021) compared to two time periods prior to the intervention: Pre-COVID-19 (09/15/2019 – 03/14/2020) and during the COVID-19 surge (03/15/2020 – 09/14/2020). The six weeks in between our intervention period and the COVID-19 surge period represents our start-up period for the intervention, also known as a "washout" period.*

Specific Aims:

Aim 1: To test the combined effects of an ACP Program on rates of ascertainable quality measures of ACP. We will conduct a Pre-Post, open-cohort design, pragmatic trial with 150 clinicians and evaluate the effectiveness of the intervention by comparing ACP documentation among ***13,000 patients over 65***. ***Hypothesis****: A higher proportion of*

patients in the intervention period (vs. control period) will have ACP documentation ***(primary trial outcome).***

Aim 2: To characterize ACP documentation rates among under-served minority groups. ***Hypothesis****: Under-served minorities in the intervention period will have fewer disparities in ACP documentation.*

Exploratory Aim: Qualitative assessment of barriers and facilitators for intervention success. *Our goal is to identify and describe clinician implementation experiences .*

IMPACT: COVID-19 poses a unique dilemma for older Americans, who must balance their desire to live against the risk of a lonely and traumatic hospital death. Clinician communication training and video decision support is a practical, evidence-based, and innovative approach to assist patients facing this most difficult of all choices. Major strengths of this proposal are: the highly experienced team making this project feasible; the present infrastructure already embedded at Northwell Health; and, the potential immediate deployment of the intervention, if successful, across the country. This work holds the promise of improving the quality of care provided to millions of Americans during the COVID-19 pandemic.

2.0 Background

SIGNIFICANCE

Epidemiology: COVID-19 disproportionately affects the elderly and those with serious illnesses such as heart disease, cancer, and dementia.(6) These diseases are common, morbid, and costly conditions, especially in people over the age of 65.(7) When COVID-19 strikes, older patients die at higher rates.(8) The surge in patients with COVID-19 poses a significant challenge to clinicians and health care institutions.

Advance care planning (ACP) in older patients needs improvement: ACP seeks to ensure that patients receive medical care consistent with their values, goals and preferences when confronted with serious illness, including COVID-19.(9, 10) ACP is the most consistent factor associated with better palliative care outcomes in patients.(11, 12) The lack of ACP is associated with greater use of aggressive interventions, more terminal hospitalizations, lower hospice use, higher health care costs, and worse bereavement outcomes.(13-18) Unfortunately, ACP completion in older patients remains inadequate.(19, 20) Furthermore, marked racial and regional disparities persist in ACP documentation.(21-26) For the ACP process to lead to optimal decisions, patients require accurate and comprehensible information about their options, and a care setting where communication needs are addressed early in their illness.(27-30) However, studies show that traditional written ACP (advance directive forms) does not effectively inform many patients, and often occurs late in the disease process.(31-36) Patient understanding may be clouded due to pain, medication, or psychological reactions.(32-44) Other common

barriers to ACP include variable quality of clinician communication, complex relationships between patients and family regarding decision making, and the inability for patients and caregivers to realistically envision future health states.(45-55) Multiple barriers, particularly related to the quality of clinician communication and the ability of patients to fully understand possible future scenarios such as COVID-19, impede the effectiveness of ACP.

Lack of effective ACP during COVID-19 has led to burdensome, costly, and often avoidable interventions: Medical technology enables an extraordinary array of possible interventions for older people. However, particularly in the setting of COVID-19, the burdens of treatment may outweigh its beneficial effects. In the elderly, even the most aggressive treatment may not prevent death from the clinical complications of COVID-19, which include respiratory failure, renal failure, and cardiac arrest.(56) Furthermore, and unique to this pandemic, hospitalized patients may not receive visitors, thus introducing loneliness and a sense of abandonment to what is already a traumatic death. Given this risk-benefit profile, many high-risk patients, particularly among the elderly, may choose to decline aggressive treatments or even hospitalization. Yet, without the presence of a documented ACP discussion, the default approach is to perform all invasive procedures. Research suggests that “rescue” interventions such as mechanical ventilation and attempted cardio-pulmonary resuscitation (CPR) are inappropriately used even when not desired.(57-59) Despite the mounting evidence suggesting that older patients with serious illness prefer palliation, they often receive burdensome interventions and sub-optimal control of pain and other distressing symptoms, particularly in the absence of high-quality ACP.(57, 60-62) The converse is true for patients with documented end-of-life decisions.(11, 12, 21, 63-66) ACP is necessary to avoid unwanted interventions; high-quality ACP increases patient safety by ensuring that patients receive effective medical care that matches their goals.

Clinician communication training and video decision support improves ACP: Traditional approaches to ACP, which often rely on ad hoc verbal descriptions of hypothetical clinical situations and treatment choices, is limited because complex scenarios are difficult to envision, provider information is inconsistent, and verbal explanations are hampered by literacy, emotional, and language barriers.(32, 67-68) Patients often do not fully understand the choices presented in advance directive legal documents, and the quality of communication about these choices is suboptimal.(69) Over the past few years, investigators have recognized the shortcomings of prior efforts and developed new interventions to better facilitate ACP.(70) The Comprehensive Telehealth ACP Program for COVID-19 proposed for this study trains patients and clinicians to communicate about ACP in the context of COVID-19. Prior work, including several RCTs, supports the efficacy of the ACP Decisions videos and the VitalTalk program in older patients.(71-87) This application represents the first Comprehensive Telehealth ACP Program for COVID-19 that combines two well-tested, evidence-based, and complementary interventions using an online platform.

Our research group’s expertise in Natural Language Processing (NLP) and ongoing U grant at Northwell Health enables a study of this scale and on a shortened timeline: For

the proposed trial, we will use NLP, a form of computer-assisted abstraction, to detect our primary outcome, ACP documentation in the EHR. Our research group has published the seminal studies using NLP in ACP, and we have already applied this methodology at Northwell Health in our ongoing study (1UH3AG060626-02). Northwell Health, the largest health care system in New York, is an ideal setting for this trial. The ability to use NLP to quickly and rapidly abstract outcomes from a large number of EHR records, and our present work with Northwell Health, lays the groundwork for this trial.

A Pre-Post, open-cohort, pragmatic trial is well-suited to evaluate an ACP Program intervention in older patients during the COVID-19 pandemic: Several notable advantages of this design exist for testing communication interventions in older patients during a pandemic.(88-91) In response to COVID-19 and the increased mortality, we expect that Northwell Health ACP activity increased naturally. Two six-month control periods prior to the wide-scale implementation of the intervention, allow us to measure a baseline rate of ACP activity and identify the “pre-COVID-19 ACP baseline rate” and a “COVID-19 ACP baseline rate.” We chose September 15, 2019 as the start of the pre-COVID-19 control period since it is about one year before the intervention start, and we chose March 15, 2020 as the start of the baseline control period since the earliest ACP activities due to COVID-19 began during that month.(92, 93) We will then compare the ACP rate during the implementation period, which begins November 1, 2020 and continues for six months, to the rates in the previous two control periods. Given that the intervention is entirely online, no logistic barriers prohibit us from training providers on a shortened timeline.(94) Clinicians will be trained during the early weeks of the trial, avoiding any contamination during the control period. In addition, the low-risk intervention being tested will be implemented at the clinic level and our ongoing study received IRB-approval exempting it from individual consent procedures when using an open-cohort design. Thus, the proposed trial is an optimal design for a COVID-19 study and a logical next step toward understanding the real-world application of the Comprehensive ACP Program. Taken together, this work has the potential to improve the quality of care provided to millions of older Americans during the COVID-19 pandemic.

3.0 Inclusion and Exclusion Criteria

Clinic Eligibility: Clinics affiliated with Northwell Health may be included in the study.

Clinician, Staff Eligibility: Any staff member identified by the site-PIs (Drs. LaVine, Carney and Burns) who are affiliated with Northwell Health.

Patient Eligibility: To be eligible for this study, individuals must be aged 65 or over, affiliated with the Northwell Health clinic. For each of the three periods of the study, those patients over the age of 65 who are engaged (e.g., seen in person, telehealth, etc.) with the clinic during that time period will be included in that time period.

- We will not be including individuals who are not yet adults (infants, children, teenagers)

- We will not be including pregnant women
- We will not be including prisoners

4.0 Study-Wide Number of Subjects*

We will study data from 13,000 patients aged 65 or older for our primary (completed advance care plans) outcome. ACP can include designating a health care proxy, discussion of advance directives, goals-of-care discussions, CPR discussions, palliative care discussions and referrals, as well as hospice. Each of these ACP outcomes will also be compared in secondary analyses using NLP. These data will be obtained from the EHR. All patient participants will only be included for medical record review.

In addition, we will recruit up to 30 clinicians, from participating clinics, who completed the intervention training. We will ask them to complete a qualitative interview assessing their experiences with the ACP intervention.

5.0 Study-Wide Recruitment Methods*

Patients will not be recruited into the study, rather we will seek a waiver of individual informed consent and HIPAA authorization since the research involves no more than minimal risk to subjects.

The 30 clinicians recruited for the qualitative interviews will participate only after providing individual verbal informed consent.

We do not foresee a significant challenge with ensuring follow-up as all patient data is collected passively via the EHR, and the clinician interviews are single episode data collections.

6.0 Multi-Site Research*

Communication Plan:

We will hold:

Bi-weekly calls with all study site RAs and other site-specific staff as necessary. The calls are overseen by Drs. James Tulsky and Angelo Volandes. Items addressed include ongoing trainings, review of research progress, alerts about protocol changes, and any other issues related to subject or research team compliance.

Monthly Data Team Calls. Discuss progress and concerns of all data related topics. These calls are run by Drs. Charlotta Lindvall and Josh Lakin.

We also have a “Communications Log” where we track the important calls, meetings and interactions.

Participating Institution
Each Participating Institution is expected to comply with all applicable federal regulations and DF/HCC requirements, the protocol and HIPAA requirements.

The general responsibilities for each Participating Institution may include but are not limited to:

- Document the delegation of research specific activities to study personnel.
    - Commit to the accrual of participants to the protocol.
    - Submit protocol and/or amendments to their local IRB of record.
    - Maintain regulatory files as per sponsor requirements.
    - Provide the Coordinating Center with regulatory documents or source documents as requested.

1. Participate in protocol training prior to enrolling participants and throughout the trial as required (i.e., teleconferences).
2. Update Coordinating Center with research staff changes on a timely basis.
3. Submit protocol deviations and violations to local IRB per institutional requirements and to the DF/HCC Sponsor in accordance with DF/HCC requirements.
4. Have office space, office equipment, and internet access that meet HIPAA standards.
5. Participate in any quality assurance activities and meet with monitors or auditors at the conclusion of a visit to review findings.
6. Promptly provide follow-up and/or corrective action plans for any monitoring queries or audit findings.

Long-term storage of Data:

Data stored on the DFCI server will reside there only for the periods they are required to be there for study usage. Data will be securely removed from these servers on a per-item basis. Removed data will be securely transferred to DFCI long-term servers for storage.

Dana Farber, Northwell Health, Boston Medical Center (BMC) and Massachusetts General Hospital (MGH) Roles:

As in the ACP PEACE Trial, Dana Farber is the IRB of record and Northwell Health has ceded IRB review to DFCI. Northwell Health will review clinical notes from the medical records for ACP document using the NLP software ClinicalRegex. The results of this NLP review will be stripped of any PHI sent to DFCI for analysis. The review of these records will be used to study the impact of the clinician trainings and patient educational videos currently being implemented at Northwell Health primary care practices on patient

advance care planning outcomes. Study staff at BMC will assess the implementation of the intervention across hospital's outpatient internal medicine clinics. BMC will receive and analyze limited data set(s). All data that BMC receives will be de-identified and there will be no PHI. At MGH, Dr. Volandes is co-leading the entire study along with Dr. Tulsky. He will oversee the implementation of the videos with NWH. No identified data or PHI will be involved in MGH's role.

The clinician interview recordings will be captured via Northwell's Teams platform. The recordings will be transcribed and Northwell will provide access to a shared drive. The shared drive will contain the de-identified interview transcriptions. There will be no transfer of data, the data will stay at Northwell.

The audio recordings will be saved in a secure HIPPA ePHI drive (OneDrive) and will be assigned a unique identifier. A professional research transcriber will then generate transcripts based on the audio recordings. The transcripts will be assigned unique identifiers and be uploaded into a secure HIPPA ePHI drive for analysis by the BMU study team. Only IRB approved study personnel will have access to the study data.

The audio recordings will be transcripted and then the recordings will be destroyed to be de-identified. We will redact the names on the transcriptions.

7.0 Study Timelines

The study has three distinct time periods: A Pre-COVID-19 baseline period (September 15, 2019 to March 14, 2020), a COVID-19 period (March 15, 2020 – September 14, 2020), and period after the implementation of the intervention (November 1, 2020 – April 30, 2021).

During the first month of the project, we will organize the logistics for study implementation and prepare NLP and other tools. By month 2 we will have organized clinician trainings—clinician re-training will then reoccur between months 3-8. Months 3-10 will include data collection, cleaning, and analysis. Manuscript preparation and dissemination of the trial results will occur during the final two months.

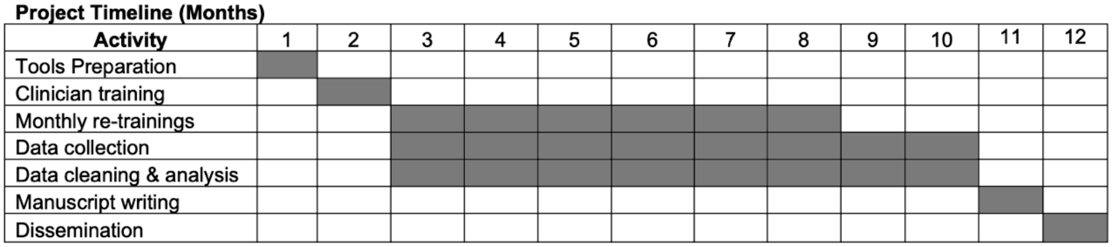

8.0 Study Endpoints

Primary and Secondary Endpoints:

We will conduct a Pre-Post, open-cohort, pragmatic trial of the Comprehensive Telehealth ACP Program to promote ACP documentation in 13,000 patients aged 65 and over affiliated with 150 outpatient primary care clinicians from Northwell Health. The primary outcome for this pragmatic trial is documentation of ACP in older patients. We will assess ACP documentation for the two periods prior to the intervention (September 15, 2019 – March 14, 2020, pre-COVID-19 baseline rate; and, March 15, 2020 – September 14, 2020, COVID-19 rate), and compare them to the six-months after the implementation of the intervention (November 1, 2020 – April 30, 2021). Our primary outcome is documentation of ACP activity as determined through NLP of EHR records confirmed by human coders. Discussions of health care proxy, advance directives, goals of care, completion of advance plans, and changes of resuscitation orders, palliative care and hospice discussions, will all count in this composite measure. These outcomes will be ascertained from the records of 13,000 patients across Northwell Health's clinics.

Secondary Safety Endpoints:
There is a risk of patients becoming uncomfortable with discussing these topics; however, ACP is routine and part of the standard of care.

9.0 Procedures Involved

The present intervention has already been IRB-approved and being successfully conducted at Northwell Health in the system's cancer clinics (1UH3AG060626-02). The present proposal expands our ongoing work from the cancer setting to the outpatient primary care setting.

A Pre-Post, open-cohort, pragmatic trial study design will compare the rate of ACP documentation during the intervention to two control periods. Two six-month control periods prior to the wide-scale implementation of the intervention, allow us to measure a baseline rate of ACP activity and identify the "pre-COVID-19 ACP baseline rate" and a "COVID-19 ACP baseline rate." We chose September 15, 2019 as the start of the pre-COVID-19 control period since it is about one year before the intervention start, and we chose March 15, 2020 as the start of the baseline control period since the earliest ACP activities due to COVID-19 began during that month. We will then compare the ACP rate during the implementation period, which begins November 1, 2020 and continues for six months, to the rates in the previous two control periods.

Data Collection Protocol:

Patients: Data will be collected from 13 months prior to the implementation start date (i.e., September 15, 2019) and continue for six months after the implementation start date (i.e., November 1, 2020 – April 30, 2021). Our primary (ACP documentation) outcome

will be abstracted from Northwell's data platform, which has real-time data for these variables, using NLP. ***We are already using the process for our ongoing trial.***

Qualitative Interviews:

Northwell will identify ~~the~~ clinicians from those who completed VitalTalk training, email the Detailed Information Sheets and facilitate scheduling the interviews. The investigator conducting the interview will review the information on the Detailed Information Sheet with the clinician on the same day as their interview.

VitalTalk communication skills training: Clinicians will receive VitalTalk intensive communication skills training via highly structured Zoom conference. They will learn skills relevant to discussions about ACP and COVID-19 including delivering serious news, eliciting goals of care, and managing difficult conversations via telehealth platforms. The training is based upon the pedagogical framework that skill acquisition requires observation, practice, and feedback. The synchronous training will be done in two two-hour or one four-hour, small group sessions where clinicians practice specific skills such as "ask-tell-ask" or responding to patient emotion and learn to follow "talking maps" that provide a guide through the conversation about goals of care. In these online sessions with simulated patient actors, the clinicians will set a learning goal, practice the skill in question, receive constructive feedback in a highly structured format, and finally have opportunities to "rewind and replay," so that they can experience success and see its effect on the patient. Participating clinicians will also receive the VitalTalk Tips smartphone app that provides daily skill reminders and access to all of the COVID-specific communication skills and will attend a one-hour refresher training webinar every month to address challenges that may have arisen and to sharpen their newly learned communication skills. This online course will teach content equivalent to the current VitalTalk Mastering Tough Communication courses that are used in multiple health care settings. ***VitalTalk has trained over 10,000 clinicians worldwide using this approach and provides the communication skills clinicians need to provide high-quality care to patients at risk or with COVID-19.***

Suite of ACP videos for COVID-19: All clinicians will also be trained remotely on the ACP Decisions Video Program using the ACP app. Training will instruct clinicians on how to: 1. Introduce the COVID-19 videos to patients and caregivers; 2. Select the appropriate video(s) from the entire suite according to patients' needs; and, 3. Prescribe videos for patients and caregivers using the electronic platform. The suite of ACP videos is designed to address common ACP decisions confronting patients at risk or with COVID-19 and their caregivers. The videos are intended to be an adjunct to clinician counseling, not to replace it. The videos range from 4-6 minutes and were developed using an iterative process of drafting and review by infectious disease experts, geriatricians, critical care doctors, palliative care clinicians, patients, and family members. The content in the videos is intended to be objective and balanced and is scripted at a sixth-grade level of health literacy or less. The videos are available in 25 languages. The videos have closed captioning, and can be viewed in real time at an office

visit with an iPad, or if done remotely (e.g., by phone or telemedicine) via a web-link. Northwell Health uses a vast network of telemedicine tools to communicate with patients including smartphones and tablet computers, which all support video viewing.

Intervention training: Remote clinician training will occur during the early weeks of the trial. Clinicians will be trained together in small groups. Training will instruct the clinicians on how to: i. more effectively communicate with patients regarding COVID-19, ii. have ACP conversations with patients, iii. introduce the videos to patients and families, iv. use the videos as an adjunct to ACP counseling by clinicians, v. select the appropriate video(s) from the entire suite as according to patients' needs, and, vi. use the app or electronic platform for prescribing videos to be seen at home (telehealth visits), or to be viewed in clinic with an iPad. The Northwell Health Telehealth program is a mature program in which the majority of patients have access to either a smartphone or tablet for virtual clinical care. The intervention training program will leverage the infrastructures and tools already developed in our ongoing research study within Northwell. Each month, one-hour refresher trainings will be available to all clinicians. We also expect the clinics to have some staff turnover and new hires over the course of the trial; thus onboarding and training will be made available on an ongoing basis.

Data collected for long-term follow-up:

Performance of NLP

Dr. Lindvall's lab at DFCI has published several studies on the use of NLP to capture documentation of ACP in clinical notes.(91 – 93) Compared to standard medical record review her team has achieved a sensitivity 97% - 99% and specificity 85% - 100% at the note level. At the patient level, both sensitivity and specificity are close to 100%. Patients accrue hundreds to thousands of notes which makes standard medical review not feasible, and this method dramatically increases efficiency and sensitivity.

Advances in computer science techniques such as NLP allow for efficient and accurate analysis of the free-text from clinical notes in order to detect our primary outcomes. The increasing use of EHR combined with computational advances in NLP can accurately quantify our primary outcome.

The below procedures will be completed at Northwell Health by the respective site-Investigators. No patient identifiable information will leave the HIPPA-secure firewall of the health care system.

Each site-Investigator will use NLP to identify the following primary outcome.

Data will be abstracted from the EHR data repository. Demographic information and baseline characteristics relevant will be collected. Analysis of free text regarding ACP will be extrapolated using computational methods.

Individual variables have been selected as they are basic demographic and baseline characteristics of study participants.

The Investigators will use computational methods (NLP) to identify our outcomes. Once these variables have been abstracted from the health care system's EHR, the data will be collected for each patient and the patient will be given a random unique identifier. A cross-walk of the unique identifier and the identifiable information of the patient will be securely stored at the health care system and not transferred to DFCI.

The deidentified data for each patient will then be sent to DFCI and MGH for further analysis.

Only researchers listed on this IRB will be able to access the data. The risks will be minimal as the data will be stored and analyzed on the HIPAA secure cluster at each health care system. None of the data will be stored in paper form. The data and identifiers will be kept for three years on the HIPPA secure cluster computer After the three years, the data will be permanently destroyed.

10.0 Data and Specimen Banking

NA

11.0 Data Management and Confidentiality

DATA ANALYSES

Aim 1: To test the combined effects of an ACP Program on rates of ascertainable quality measures of ACP among 13,000 patients at risk or with COVID-19. We will use an intention to treat approach. All patients over 65 engaged with the clinic over the course of each time period in each of the three study periods will be included in the analysis. We will use generalized linear mixed models to compare outcomes between intervention and the two control periods. The basic model is depicted in Equation (1)

$g(Y_{ijk}) = \mu + \alpha_i + \beta_j + \gamma_k + X_{ik}\theta$ (Eq 1)

where Y_{ijk} denotes the response from individual *k* from cluster (PCP) *j* at period *i*. The model includes a fixed effect α_i for period *i*, which indicates the difference between periods. Our primary comparison is between the post-intervention period and the two control periods. The model also includes a random effect β_j for cluster *j* to take into account the patients within PCP clustering. It is likely a large proportion of patients will overlap in the adjacent study periods; therefore, the model includes a random subject effect γ_k for individual *k* to take into account the repeated measures from the same subject. We will limit the analysis to those who appear in two or more comparison

periods as a secondary analysis. The model also includes an additional term of X_{ik} which represents the patient characteristics at period *i* to adjust for potential confounding factors. We will use a logit link (g) for the binary outcomes which include our primary outcome of ACP documentation.

Sample Size Calculation & Power Analysis: We estimated approximately 7,800 patients from 150 PCPs are eligible for the study at each period and 85% patients overlap from one period to the next. With 3 time periods, we anticipate a total of 10,139 unique patients will be included in the study. For our primary comparison between COVID-19 period and post intervention period, we expect 7,800 patients in each of the two periods with 6,630 appearing in both periods. In the most conservative scenario with each PCP contributing an average 44 patients, the design effect is 3.2 assuming an intra-cluster correlation of 0.05, which corresponds to an effective sample size of 2,098 appearing in both periods. Preliminary estimates indicate the rate for ACP documentation (primary outcome) is around 10% pre-COVID and 20% during COVID. The study will have more than 95% power to detect a 5% absolute increase in outcome with a two-sided significance level of 0.05. The power will be even higher for a 10% post intervention increase, which is our threshold of a clinically meaningful difference.

Exploratory Aim: To examine facilitators and barriers to intervention success, we will conduct a qualitative analysis of interview transcripts collected from 30 participating clinicians. An examination of their perceptions of the training and video intervention, as well as facilitators and barriers to implementation will help to contextualize, and possibly explain, the trial findings.

Aim 2: To characterize ACP documentation rates among under-represented minority groups. The analysis will use a difference in differences approach comparing the changes from the control period to the intervention period between White and non-White racial minority and between non-Medicaid and Medicaid patients. This will translate into adding a subgroup fixed effect to (Eq 1) and test the interaction between period and subgroups. We will explore the intervention effect in each racial minority (Black, Hispanic, Asian) separately.

Sample Size Calculation & Power Analysis: Preliminary estimates indicate 30% of the population is non-white racial minority and approximately 30% of the population were on Medicaid. Assuming the rate for ACP documentation is 21.5% among Whites and 16.5% among non-White (overall 20%) during COVID, and both groups increase to 30% post-intervention, our simulation study shows that we will have >90% power to detect a 5% difference in differences after taking into account of clustering. We anticipate a larger difference in differences between Medicaid and non-Medicaid patients; therefore, the power for that analysis will be even higher.

SECURING THE DATA
EHR Data Transfer Procedures

1. Representatives from Northwell Health will make six-month data "dumps", using appropriately secure tools, of EHR data onto selected servers.
2. Scheduled job run by DFCI will initiate the ingestion of new data as made available.
3. DFCI's system manager replaces person identifiers, including all HIPAA face identifiers, with an alphanumeric ID.

12.0 Provisions to Monitor the Data to Ensure the Safety of Subjects*

NA

13.0 Withdrawal of Subjects

"Broadcast notifications":

We will be providing any potential patients (participants) with the opportunity to opt out of this project entirely (i.e., to have their deidentified data from the EHR not included in our main analysis). We have created "broadcast notifications" in the form of a poster in the clinic/patient areas giving brief commentary about the trial and their choice to participate or not to participate in having their data included in our analysis.

14.0 Risks to Subjects

Potential Risks

The potential risks are minimal given the fact that the intervention promotes learning about ACP, improves communication for patients and their families regarding ACP and self-determination, and the concordance between patient's wishes and the care they receive.

The major potential risk for subjects is loss of confidentiality. Loss of confidentiality is very unlikely because specific procedures have been implemented by the research team to prevent such disclosure and these measures will be maintained during the proposed study.

Another risk is being upset by the questions, which are part of the standard of care. Probability of occurrence is minimal. We have conducted a series of clinical trials for patients with advance illnesses and have rarely had patients get upset due to the topic. In each of these cases, the participant was interested in continuing after a short break.

Clinicians may find some of the interview content difficult as we will be discussing their experience of advance care planning with patients, some of whom may have been very

sick or died from COVID. During the interview we will stop at their request or skip questions that make them uncomfortable. If needed, we can recommend that they contact their employee wellness or Mental Health program.

Protects Against Risk:

We will emphasize that participation in this research is voluntary. Furthermore, Northwell Health allows patients to decline having their deidentified data including for research purposes.

This study includes data from hospital data systems. We will access patient records for research study data within the explicit guidelines of the limited data specified.

Risks to confidentiality of the data collected throughout the proposed study will be addressed as follows: all information in the database will be indexed by subject identifier only, so that even if the database server is compromised subjects cannot be identified, thus maintaining the privacy of their information. Also, assurance and confidentiality of information will be made to all subjects. Data will be handled with the same confidentiality accorded to patient's medical records. Specific procedures protecting subject confidentiality will be as follows:

- Access to data files will be secured with a password-filing system (that logs entry) and is restricted to authorized staff only.
- Necessary hard-copy records containing study data of any type will be kept in locked files.
- Master lists linking subject information with ID number will be numbered consecutively and prepared before data collection (to ensure accurate accounting). These lists will be kept locked, in duplicate, with access only by the PIs and the other investigators.
- All project staff will sign an oath of confidentiality to ensure their understanding of the terms of confidentiality required. They will be trained in specific procedures to ensure confidentiality.
- Sign-out procedures for all access to data files will be strictly enforced.
- All reports and publications will preserve the subjects' anonymity.

Any breach of confidentiality will be subject to a root cause analysis and preventive measures taken as appropriate. All of these procedures are likely to be effective based on our prior research experience.

15.0 Potential Benefits to Subjects

Potential Benefits of the Proposed Research to Human Subjects and Others

There is the potential for patients and clinicians in the clinics to benefit from the study by having their treatments better aligned with their preferences. Society, medical science and the health care system may benefit from the information obtained in the research on

Importance of the Knowledge to be Gained

There is the potential to validate an intervention that could ensure that treatments are better aligned with patients' preferences. The minor risks for the participants in this study may be considered counterbalanced by the potential direct benefits and knowledge gained. The results gleaned from the study are intended to improve the ACP of the overall outpatient clinic population. Thus, the risk/benefit balance for this study appears favorable.

16.0 Sharing of Results with Subjects

The results will be shared via a patient friendly pamphlet that explains the results of the study and is disseminated in participating clinics. An e-version will also be available on any websites affiliated with the practice.

17.0 Setting

Location:
Patients from the Northwell Health clinics will be enrolled.

Eligibility Estimates:
We anticipated that a total of 150 clinicians to be included in the trial.

Qualifications
James A. Tulsky, MD, Co-**Principal Investigator:** Dr. Tulsky is Professor of Medicine at Harvard Medical School and Chair, Department of Psychosocial Oncology and Palliative Care at DFCI. He has led multiple federally funded studies examining patient-provider communication, and is a Founding Director of VitalTalk, a non-profit organization devoted to teaching physicians communication skills (www.VitalTalk.org). Dr. Tulsky receives no compensation from VitalTalk. Dr. Tulsky will provide scientific leadership over all aspects of the study. He will oversee the conduct of the study including protocol development, training of research staff, codebook development, data analysis and manuscript writing.

Angelo Volandes, MD, MPH, Co-Principal Investigator. Dr. Volandes is a general internist and clinical researcher with past training in medicine, decision-making, bioethics, and film-making. His research career has been dedicated to developing and evaluating video decision support tools for life-limiting conditions. He currently leads an innovative team of video decision scientists at the Massachusetts General Hospital. He has successfully led many studies funded by the NIH evaluating the efficacy of these tools, the results of which have been published in top-tier journals.

Feasibility:

- The present methodology has already been IRB-approved and being successfully conducted at Northwell Health in the system's cancer clinics (1UH3AG060626-02). The present proposal expands our ongoing work from the cancer setting to the outpatient primary care setting. Thus, the timeline of the project is quite feasible and meets the requirements of the one-year grant.
- **Our research group's expertise in NLP and ongoing U grant at Northwell Health enables a study of this scale and on a shortened timeline:** For the proposed trial, we will use NLP, a form of computer-assisted abstraction, to detect our primary outcome, ACP documentation in the EHR. Our research group has published the seminal studies using NLP in ACP, and we have already applied this methodology at Northwell Health in our ongoing study (1UH3AG060626-02). ***The ability to use NLP to quickly and rapidly abstract outcomes from a large number of EHR records, and our present work with Northwell Health, lays the groundwork for this trial.***

18.0 Prior Approvals

NA

19.0 Recruitment Methods

All subjects engaged with the clinic will be recruited. No particular recruitment methods will be used.

20.0 Local Number of Subjects

There will be no local subjects recruited for this project.

21.0 Provisions to Protect the Privacy Interests of Subjects

The risks in this study are minimal and non-medical in nature. The primary risk is loss of confidentiality. To minimize the likelihood of a breach, we will collect only electronic data and anonymous data when possible. Only the minimum amount of PHI necessary will be collected from study participants, including clinicians and patients, and all data will be transmitted via secure, institutionally approved methods. We will emphasize that participation in this research is voluntary.

Qualitative interviews with the 30 clinicians will be conducted by personnel who have no affiliation to the clinicians' employer. Individual data from these interviews will not be shared with Northwell Health management. The transcripts will be deidentified, analyzed in composite, and only seen when published in this form in the medical literature.

We will transmit no identifiable data back to the clinic sites to avoid any risk of retribution, retaliation, or adverse consequence to disclosures which may come about through this process. Research staff, investigators, data management staff, and those with responsibility for analyses will have access to subject identifiers and private health information.

Oversight and Protocol Compliance: All research personnel will participate in human subjects training, an annual booster training, one-time data integrity and security training, and training on the protocol, which will include simulation and practice for all protocol procedures including obtaining informed consent and confirming comprehension. We will have regular team meetings and regular surveying reliability checks to ensure protocol compliance. The procedures described here address our efforts to minimize the risk of breach of confidentiality and to ensure that procedures remain in place for the protection of human subjects. All data management will be conducted at DFCI. The Center's researchers and staff have many years of experience working with similar data files.

Adverse Event Reporting: This study presents minimal risk to participants, and there are no adverse events given that ACP is part of the standard of care. Nonetheless, the PIs will monitor and report unforeseen adverse events to the DFCI IRB. If there are any concerns about privacy, will be refer the patient to the DFCI Privacy Officer, and we will refer any significant complaints to the DFCI Office of Research Studies.

22.0 Consent Process

Consent

(1) Patients (via EHR): There are special informed consent considerations in this pragmatic Pre-Post trial: the intervention is of low risk and will be implemented as the standard of care for the whole clinical unit, and data for our primary outcome and related outcomes derived from the EHR is ascertained from existing sources. Thus for this aspect of our proposal, we will seek a waiver of individual informed consent and HIPAA authorization after careful review of the criteria to do so. The research involves no more than minimal risk to the subjects as described above. We do not believe the waiver will adversely affect the rights and welfare of the subjects. As a pragmatic trial of thousands of patients, this research could not practicably be carried out without the waiver nor without access to and use of PHI of patients. Finally, we have developed a plan to protect identifiers from improper use and disclosure. It is our assessment that this study meets the regulatory requirements for the preparatory for research provisions of the HIPAA privacy rule and will work with the institutional IRB and Privacy Officer to establish that this does meet the standards for the security of protected health information. In accordance with this provision, only researchers and staff who are a part of the "covered entity" will use the preparatory research provision to contact prospective research subjects and no identifiable PHI will leave any covered entity without consent and an approved

procedure. We are currently conducting a study with similar EHR data abstraction via NLP and have been granted a waiver of consent and a HIPAA waiver for these activities.

(2) Clinicians (Qualitative Interviews): We will obtain individual verbal informed consent from all clinicians research subjects. While clinician subjects will be presented with all required elements of consent via a Detailed Information Sheet that is (emailed, mailed, described over the phone, etc), a signed document will not be obtained.

Non-English Speaking Subjects = N/A
Subjects who are not yet adults (infants, children, teenagers) = N/A
Cognitively Impaired Adults = N/A
Adults Unable to Consent = N/A

1.DATA OBTAINED FROM THE EHR:
To obtain personal health information from the EHRs, a waiver of HIPAA authorization will be sought.

2. DATA OBTAINED FROM CLINICIANS THROUGH QUALITATIVE INTERVIEW:
For the clinician participants (N=30) being interviewed to answer the exploratory aim about implementation, verbal informed consent will be obtained.

1. El-Jawahri A, Podgurski LM, Eichler AF, Plotkin SR, Temel JS, Mitchell SL, Chang Y, Barry MJ, Volandes AE. Use of video to facilitate end-of-life discussions with patients with cancer: a randomized controlled trial. Journal of clinical oncology : official journal of the American Society of Clinical Oncology. 2010;28(2):305-10. Epub 2009/12/02. doi: 10.1200/jco.2009.24.7502. PubMed PMID: 19949010; PMCID: PMC3040012.
2. Volandes AE, Paasche-Orlow MK, Mitchell SL, El-Jawahri A, Davis AD, Barry MJ, Hartshorn KL, Jackson VA, Gillick MR, Walker-Corkery ES, Chang Y, Lopez L, Kemeny M, Bulone L, Mann E, Misra S, Peachey M, Abbo ED, Eichler AF, Epstein AS, Noy A, Levin TT, Temel JS. Randomized controlled trial of a video decision support tool for cardiopulmonary resuscitation decision making in advanced cancer. Journal of clinical oncology : official journal of the American Society of Clinical Oncology. 2013;31(3):380-6. Epub 2012/12/13. doi: 10.1200/jco.2012.43.9570. PubMed PMID: 23233708; PMCID: PMC4090424.
3. El-Jawahri A, Mitchell SL, Paasche-Orlow MK, Temel JS, Jackson VA, Rutledge RR, Parikh M, Davis AD, Gillick MR, Barry MJ, Lopez L, Walker-Corkery ES, Chang Y, Finn K, Coley C, Volandes AE. A Randomized Controlled Trial of a CPR and Intubation Video Decision Support Tool for Hospitalized Patients. Journal of general internal medicine. 2015;30(8):1071-80. Epub 2015/02/19. doi: 10.1007/s11606-015-3200-2. PubMed PMID: 25691237; PMCID: PMC4510229.
4. Back AL, Arnold RM, Baile WF, Fryer-Edwards KA, Alexander SC, Barley GE, Gooley TA, Tulsky JA. Efficacy of communication skills training for giving bad news and discussing transitions to palliative care. Archives of internal medicine. 2007;167(5):453-60. Epub 2007/03/14. doi: 10.1001/archinte.167.5.453. PubMed PMID: 17353492.
5. Tulsky JA, Arnold RM, Alexander SC, Olsen MK, Jeffreys AS, Rodriguez KL, Skinner CS, Farrell D, Abernethy AP, Pollak KI. Enhancing communication between oncologists and patients with a computer- based training program: a randomized trial. Annals of internal medicine. 2011;155(9):593-601. Epub 2011/11/02. doi: 10.7326/0003-4819-155-9-201111010-00007. PubMed PMID: 22041948; PMCID: PMC3368370.
6. Berger NA, Savvides P, Koroukian SM, Kahana EF, Deimling GT, Rose JH, Bowman KF, Miller RH. Cancer in the elderly. Transactions of the American Clinical and Climatological Association. 2006;117:147-55; discussion 55-6. Epub 2008/06/06. PubMed PMID: 18528470; PMCID: PMC1500929.
7. Howlader N, Noone AM, Krapcho M, Miller D, Bishop K, Altekruse SF, Kosary CL, Yu M, Ruhl J, Tatalovich Z, Mariotto A, Lewis DR, Chen HS, Feuer EJ, Cronin KA (eds). SEER Cancer Statistics Review, 1975-2013, National Cancer Institute. Bethesda, MD, https://seer.cancer.gov/csr/1975_2013/, based on November 2015 SEER data submission, posted to the SEER web site, April 2016.
8. Yabroff KR, Lamont EB, Mariotto A, Warren JL, Topor M, Meekins A, Brown ML. Cost of care for elderly cancer patients in the United States. Journal of the National Cancer Institute. 2008;100(9):630- 41. Epub 2008/05/01. doi: 10.1093/jnci/djn103. PubMed PMID: 18445825.
9. Tulsky JA. Improving quality of care for serious illness: findings and recommendations of the Institute of Medicine report on dying in America. JAMA

internal medicine. 2015;175(5):840-1. Epub 2015/03/03. doi: 10.1001/jamainternmed.2014.8425. PubMed PMID: 25730201.
10. Aitken PV, Jr. Incorporating advance care planning into family practice [see comment]. American family physician. 1999;59(3):605-14, 17-20. Epub 1999/02/25. PubMed PMID: 10029787.
11. Mack JW, Weeks JC, Wright AA, Block SD, Prigerson HG. End-of-life discussions, goal attainment, and distress at the end of life: predictors and outcomes of receipt of care consistent with preferences. Journal of clinical oncology : official journal of the American Society of Clinical Oncology. 2010;28(7):1203-8. Epub 2010/02/04. doi:10.1200/jco.2009.25.4672. PubMed PMID: 20124172; PMCID: Pmc2834470.
12. Wright AA, Zhang B, Ray A, Mack JW, Trice E, Balboni T, Mitchell SL, Jackson VA, Block SD, Maciejewski PK, Prigerson HG. Associations between end-of-life discussions, patient mental health, medical care near death, and caregiver bereavement adjustment. JAMA : the journal of the American Medical Association. 2008;300(14):1665-73. Epub 2008/10/09. doi: 10.1001/jama.300.14.1665. PubMed PMID: 18840840; PMCID Pmc2853806.
13. Detering KM, Hancock AD, Reade MC, Silvester W. The impact of advance care planning on end of life care in elderly patients: randomised controlled trial. BMJ (Clinical research ed). 2010;340:c1345. Epub 2010/03/25. doi: 10.1136/bmj.c1345. PubMed PMID: 20332506; PMCID: Pmc2844949.
14. U.S. Department of Health and Human Services; Office of the Assistant Secretary for 2. Planning and Evaluation; Office of Disability, Aging and Long-Term Care Policy. Advance directives and advance care planning: Report to Congress. 2008. http://aspe.hhs.gov/daltcp/reports/2008/ADCongRpt.pdf. Accessed May 25, 2017.
15. Mitchell SL, Kiely DK, Hamel MB. Dying with advanced dementia in the nursing home. Archives of internal medicine. 2004;164(3):321-6. Epub 2004/02/11. doi: 10.1001/archinte.164.3.321. PubMed PMID: 14769629.
16. Mitchell SL, Teno JM, Kiely DK, Shaffer ML, Jones RN, Prigerson HG, Volicer L, Givens JL, Hamel MB. The clinical course of advanced dementia. The New England journal of medicine. 2009;361(16):1529-38. Epub 2009/10/16. doi: 10.1056/NEJMoa0902234. PubMed PMID: 19828530; PMCID: Pmc2778850.
17. Bernacki RE, Block SD. Communication About Serious Illness Care Goals: A Review and Synthesis of Best Practices. JAMA internal medicine. 2014. Epub 2014/10/21. doi: 10.1001/jamainternmed.2014.5271. PubMed PMID: 25330167.
18. Thorne SE, Bultz BD, Baile WF. Is there a cost to poor communication in cancer care?: a critical review of the literature. Psycho-oncology. 2005;14(10):875-84; discussion 85-6. Epub 2005/10/04. doi: 10.1002/pon.947. PubMed PMID: 16200515.
19. Heyland DK, Barwich D, Pichora D, Dodek P, Lamontagne F, You JJ, Tayler C, Porterfield P, Sinuff T, Simon J. Failure to engage hospitalized elderly patients and their families in advance care planning. JAMA internal medicine. 2013;173(9):778-87. Epub 2013/04/03. doi: 10.1001/jamainternmed.2013.180. PubMed PMID: 23545563.
20. Teno JM, Branco KJ, Mor V, Phillips CD, Hawes C, Morris J, Fries BE. Changes in advance care planning in nursing homes before and after the patient Self-

Determination Act: report of a 10-state survey. Journal of the American Geriatrics Society.1997;45(8):939-44. Epub 1997/08/01. PubMed PMID: 9256845.

21. Mitchell SL, Teno JM, Intrator O, Feng Z, Mor V. Decisions to forgo hospitalization in advanced dementia: a nationwide study. Journal of the American Geriatrics Society. 2007;55(3):432-8. Epub 2007/03/08. doi: 10.1111/j.1532-5415.2007.01086.x. PubMed PMID: 17341248.
22. White DB, Arnold RM. The evolution of advance directives. JAMA : the journal of the American Medical Association. 2011;306(13):1485-6. Epub 2011/10/06. doi: 10.1001/jama.2011.1430. PubMed PMID: 21972313.
23. Mystakidou K, Parpa E, Tsilila E, Katsouda E, Vlahos L. Cancer information disclosure in different cultural contexts. Supportive care in cancer : official journal of the Multinational Association of Supportive Care in Cancer. 2004;12(3):147-54. Epub 2004/04/13. doi: 10.1007/s00520-003-0552-7. PubMed PMID: 15074312.
24. Johnson KS, Kuchibhatla M, Tulsky JA. What explains racial differences in the use of advance directives and attitudes toward hospice care? Journal of the American Geriatrics Society. 2008;56(10):1953-8. Epub 2008/09/06. doi: 10.1111/j.1532-5415.2008.01919.x. PubMed PMID: 18771455; PMCID: PMC2631440.
25. Mack JW, Paulk ME, Viswanath K, Prigerson HG. Racial disparities in the outcomes of communication on medical care received near death. Archives of internal medicine. 2010;170(17):1533-40. Epub 2010/09/30. doi: 10.1001/archinternmed.2010.322. PubMed PMID: 20876403; PMCID: PMC3739279.
26. Smith AK, McCarthy EP, Paulk E, Balboni TA, Maciejewski PK, Block SD, Prigerson HG. Racial and ethnic differences in advance care planning among patients with cancer: impact of terminal illness acknowledgment, religiousness, and treatment preferences. Journal of clinical oncology : official journal of the American Society of Clinical Oncology. 2008;26(25):4131-7. Epub 2008/09/02. doi: 10.1200/jco.2007.14.8452. PubMed PMID: 18757326; PMCID: PMC2654372.
27. Emanuel LL, von Gunten CF, Ferris FD. Advance care planning. Archives of family medicine. 2000;9(10):1181-7. Epub 2000/12/15. PubMed PMID: 11115227.
28. Emanuel LL, Barry MJ, Stoeckle JD, Ettelson LM, Emanuel EJ. Advance directives for medical care--a case for greater use. The New England journal of medicine. 1991;324(13):889-95. Epub 1991/03/28. doi: 10.1056/nejm199103283241305. PubMed PMID: 2000111.
29. U.S. Department of Health and Human Services; Office of the Assistant Secretary for 2. Planning and Evaluation; Office of Disability, Aging and Long-Term Care Policy. Advance directives and advance care planning: Report to Congress. 2008.http://aspe.hhs.gov/daltcp/reports/2008/ADCongRpt.pdf. Accessed May 25, 2017.
30. Morrison RS, Meier DE. High rates of advance care planning in New York City's elderly population. Archives of internal medicine. 2004;164(22):2421-6. Epub 2004/12/15. doi: 10.1001/archinte.164.22.2421. PubMed PMID: 15596631.

31. Fagerlin A, Schneider CE. Enough. The failure of the living will. The Hastings Center report. 2004;34(2):30-42. Epub 2004/05/26. PubMed PMID: 15156835.
32. Loewenstein G. Hot-cold empathy gaps and medical decision making. Health psychology : official journal of the Division of Health Psychology, American Psychological Association. 2005;24(4s):S49-56. Epub 2005/07/28. doi: 10.1037/0278-6133.24.4.s49. PubMed PMID: 16045419.
33. Billings JA. The need for safeguards in advance care planning. Journal of general internal medicine. 2012;27(5):595-600. Epub 2012/01/13. doi: 10.1007/s11606-011-1976-2. PubMed PMID: 22237664; PMCID: Pmc3326115.
34. Billings JA, Bernacki R. Strategic targeting of advance care planning interventions: the Goldilocks phenomenon. JAMA internal medicine. 2014;174(4):620-4. Epub 2014/02/05. doi: 10.1001/jamainternmed.2013.14384. PubMed PMID: 24493203.
35. Lakin JR, Block SD, Billings JA, Koritsanszky LA, Cunningham R, Wichmann L, Harvey D, Lamey J, Bernacki RE. Improving Communication About Serious Illness in Primary Care: A Review. JAMA internal medicine. 2016. Epub 2016/07/12. doi: 10.1001/jamainternmed.2016.3212. PubMed PMID: 27398990.
36. Winter L, Parks SM, Diamond JJ. Ask a different question, get a different answer: why living wills are poor guides to care preferences at the end of life. Journal of palliative medicine. 2010;13(5):567-72. Epub 2010/04/10. doi: 10.1089/jpm.2009.0311. PubMed PMID: 20377499; PMCID: PMC2938890.
37. Bernacki RE, Block SD. Communication About Serious Illness Care Goals: A Review and Synthesis of Best Practices. JAMA internal medicine. 2014. Epub 2014/10/21. doi: 10.1001/jamainternmed.2014.5271. PubMed PMID: 25330167.
38. Billings JA, Bernacki R. Strategic targeting of advance care planning interventions: the Goldilocks phenomenon. JAMA internal medicine. 2014;174(4):620-4. Epub 2014/02/05. doi: 10.1001/jamainternmed.2013.14384. PubMed PMID: 24493203.
39. Patel K, Janssen DJ, Curtis JR. Advance care planning in COPD. Respirology (Carlton, Vic). 2012;17(1):72-8. Epub 2011/10/20. doi: 10.1111/j.1440-1843.2011.02087.x. PubMed PMID: 22008225.
40. Fagerlin A, Schneider CE. Enough. The failure of the living will. The Hastings Centerreport. 2004;34(2):30-42. Epub 2004/05/26. PubMed PMID: 15156835.
41. Davison SN, Simpson C. Hope and advance care planning in patients with end stage renal disease: qualitative interview study. BMJ (Clinical research ed). 2006;333(7574):886. Epub 2006/09/23. doi: 10.1136/bmj.38965.626250.55. PubMed PMID: 16990294; PMCID: Pmc1626305.
42. Quill TE. Perspectives on care at the close of life. Initiating end-of-life discussions with seriously ill patients: addressing the "elephant in the room". JAMA : the journal of the American Medical Association. 2000;284(19):2502-7. Epub 2000/11/14. PubMed PMID: 11074781.
43. Sharman SJ, Garry M, Jacobsen JA, Loftus EF, Ditto PH. False memories for end-of-life decisions. Health psychology : official journal of the Division of Health Psychology, American Psychological Association. 2008;27(2):291-6. Epub 2008/04/02. doi: 10.1037/0278-6133.27.2.291. PubMed PMID: 18377150.

44. Fischer GS, Tulsky JA, Rose MR, Siminoff LA, Arnold RM. Patient knowledge and physician predictions of treatment preferences after discussion of advance directives. Journal of general internal medicine. 1998;13(7):447-54. Epub 1998/08/01. PubMed PMID: 9686710; PMCID: PMC1496983.
45. De Vleminck A, Houttekier D, Pardon K, Deschepper R, Van Audenhove C, Vander Stichele R, Deliens L. Barriers and facilitators for general practitioners to engage in advance care planning: a systematic review. Scandinavian journal of primary health care. 2013;31(4):215-26. Epub 2013/12/05. doi: 10.3109/02813432.2013.854590. PubMed PMID: 24299046; PMCID: Pmc3860298.
46. De Vleminck A, Pardon K, Beernaert K, Deschepper R, Houttekier D, Van Audenhove C, Deliens L, Vander Stichele R. Barriers to advance care planning in cancer, heart failure and dementia patients: a focus group study on general practitioners' views and experiences. PloS one. 2014;9(1):e84905. Epub 2014/01/28. doi: 10.1371/journal.pone.0084905. PubMed PMID: 24465450; PMCID: Pmc3897376.
47. Ahluwalia SC, Bekelman DB, Huynh AK, Prendergast TJ, Shreve S, Lorenz KA. Barriers and Strategies to an Iterative Model of Advance Care Planning Communication. The American journal of hospice & palliative care. 2014. Epub 2014/07/06. doi: 10.1177/1049909114541513. PubMed PMID: 24988894.
48. Evans LR, Boyd EA, Malvar G, Apatira L, Luce JM, Lo B, White DB. Surrogate decision-makers' perspectives on discussing prognosis in the face of uncertainty. American journal of respiratory and critical care medicine. 2009;179(1):48-53. Epub 2008/10/22. doi: 10.1164/rccm.200806-969OC. PubMed PMID: 18931332; PMCID: Pmc2615661.
49. Fried TR, O'Leary J, Van Ness P, Fraenkel L. Inconsistency over time in the preferences of older persons with advanced illness for life-sustaining treatment. Journal of the American Geriatrics Society. 2007;55(7):1007-14. Epub 2007/07/05. doi: 10.1111/j.1532-5415.2007.01232.x. PubMed PMID: 17608872; PMCID: Pmc1948955.
50. Keating NL, Landrum MB, Rogers SO, Jr., Baum SK, Virnig BA, Huskamp HA, Earle CC, Kahn KL. Physician factors associated with discussions about end-of-life care. Cancer. 2010;116(4):998-1006. Epub 2010/01/13. doi: 10.1002/cncr.24761. PubMed PMID: 20066693; PMCID: PMC2819541.
51. Morrison RS, Morrison EW, Glickman DF. Physician reluctance to discuss advance directives. An empiric investigation of potential barriers. Archives of internal medicine. 1994;154(20):2311-8. Epub 1994/10/24. PubMed PMID: 7944853.
52. Smith AK, Williams BA, Lo B. Discussing overall prognosis with the very elderly. The New England journal of medicine. 2011;365(23):2149-51. Epub 2011/12/14. doi: 10.1056/NEJMp1109990. PubMed PMID: 22150033; PMCID: Pmc3760676.
53. Zier LS, Sottile PD, Hong SY, Weissfield LA, White DB. Surrogate decision makers' interpretation of prognostic information: a mixed-methods study. Annals of internal medicine. 2012;156(5):360-6. Epub 2012/03/07. doi: 10.7326/0003-

4819-156-5-201203060-00008. PubMed PMID: 22393131; PMCID: PMC3530840.
54. Shalowitz DI, Garrett-Mayer E, Wendler D. The accuracy of surrogate decision makers: a systematic review. Archives of internal medicine. 2006;166(5):493-7. Epub 2006/03/15.doi: 10.1001/archinte.166.5.493. PubMed PMID: 16534034.
55. Sudore RL, Schillinger D, Knight SJ, Fried TR. Uncertainty about advance care planning treatment preferences among diverse older adults. Journal of health communication. 2010;15 Suppl 2:159-71. Epub 2010/09/29. doi: 10.1080/10810730.2010.499982. PubMed PMID: 20845201.
56. Givens JL, Kiely DK, Carey K, Mitchell SL. Healthcare proxies of nursing home residents with advanced dementia: decisions they confront and their satisfaction with decision-making. Journal of the American Geriatrics Society. 2009;57(7):1149-55. Epub 2009/06/03. doi: 10.1111/j.1532-5415.2009.02304.x. PubMed PMID: 19486200; PMCID: PMC2796114.
57. Teno JM, Clarridge BR, Casey V, Welch LC, Wetle T, Shield R, Mor V. Family perspectives on end-of-life care at the last place of care. JAMA : the journal of the American Medical Association. 2004;291(1):88-93. Epub 2004/01/08. doi: 10.1001/jama.291.1.88. PubMed PMID: 14709580.
58. Mack JW, Paulk ME, Viswanath K, Prigerson HG. Racial disparities in the outcomes of communication on medical care received near death. Archives of internal medicine. 2010;170(17):1533-40. Epub 2010/09/30. doi: 10.1001/archinternmed.2010.322. PubMed PMID: 20876403; PMCID: PMC3739279.
59. Kelley AS, Ettner SL, Morrison RS, Du Q, Wenger NS, Sarkisian CA. Determinants of medical expenditures in the last 6 months of life. Annals of internal medicine. 2011;154(4):235-42. Epub 2011/02/16. doi: 10.7326/0003-4819-154-4-201102150 00004. PubMed PMID: 21320939; PMCID: PMC4126809.
60. Teno JM, Gozalo PL, Bynum JP, Leland NE, Miller SC, Morden NE, Scupp T, Goodman DC, Mor V. Change in end-of-life care for Medicare beneficiaries: site of death, place of care, and health care transitions in 2000, 2005, and 2009. JAMA : the journal of the American Medical Association. 2013;309(5):470-7. Epub 2013/02/07. doi: 10.1001/jama.2012.207624. PubMed PMID: 23385273; PMCID: Pmc3674823.
61. A controlled trial to improve care for seriously ill hospitalized patients. The study to understand prognoses and preferences for outcomes and risks of treatments (SUPPORT). The SUPPORT Principal Investigators. JAMA : the journal of the American Medical Association. 1995;274(20):1591-8. Epub 1995/11/22. PubMed PMID: 7474243.
62. Smedira NG, Evans BH, Grais LS, Cohen NH, Lo B, Cooke M, Schecter WP, Fink C, Epstein-Jaffe E, May C, et al. Withholding and withdrawal of life support from the critically ill. The New England journal of medicine. 1990;322(5):309-15. Epub 1990/02/01. doi: 10.1056/nejm199002013220506. PubMed PMID: 2296273.
63. Detering KM, Hancock AD, Reade MC, Silvester W. The impact of advance care planning on end of life care in elderly patients: randomised controlled trial. BMJ

(Clinical research ed). 2010;340:c1345. Epub 2010/03/25. doi: 10.1136/bmj.c1345. PubMed PMID: 20332506; PMCID: Pmc2844949.
64. Leung JM, Udris EM, Uman J, Au DH. The effect of end-of-life discussions on perceived quality of care and health status among patients with COPD. Chest. 2012;142(1):128-33. Epub 2012/01/14. doi: 10.1378/chest.11-2222. PubMed PMID: 22241761; PMCID: Pmc3418855.
65. Abel J, Pring A, Rich A, Malik T, Verne J. The impact of advance care planning of place of death, a hospice retrospective cohort study. BMJ supportive & palliative care. 2013;3(2):168-73. Epub 2013/04/30. doi: 10.1136/bmjspcare-2012-000327. PubMed PMID: 23626905; PMCID: Pmc3632964.
66. Silveira MJ, Kim SY, Langa KM. Advance directives and outcomes of surrogate decision making before death. The New England journal of medicine. 2010;362(13):1211-8. Epub 2010/04/02. doi: 10.1056/NEJMsa0907901. PubMed PMID: 20357283; PMCID: Pmc2880881.
67. Berkman ND, Sheridan SL, Donahue KE, Halpern DJ, Viera A, Crotty K, Holland A, Brasure M, Lohr KN, Harden E, Tant E, Wallace I, Viswanathan M. Health literacy interventions and outcomes: an updated systematic review. Evidence report/technology assessment. 2011(199):1-941. Epub 2011/03/01. PubMed PMID: 23126607; PMCID: PMC4781058.
68. Waite KR, Federman AD, McCarthy DM, Sudore R, Curtis LM, Baker DW, Wilson EA, Hasnain-Wynia R, Wolf MS, Paasche-Orlow MK. Literacy and race as risk factors for low rates of advance directives in older adults. Journal of the American Geriatrics Society. 2013;61(3):403-6. Epub 2013/02/06. doi: 10.1111/jgs.12134. PubMed PMID: 23379361; PMCID: PMC3891653.
69. Tulsky JA, Fischer GS, Rose MR, Arnold RM. Opening the black box: how do physicians communicate about advance directives? Annals of internal medicine. 1998;129(6):441-9. Epub 1998/09/12. PubMed PMID: 9735081.
70. Tulsky JA. Improving quality of care for serious illness: findings and recommendations of the Institute of Medicine report on dying in America. JAMA internal medicine. 2015;175(5):840-1. Epub 2015/03/03. doi: 10.1001/jamainternmed.2014.8425. PubMed PMID: 25730201.
71. El-Jawahri A, Podgurski LM, Eichler AF, Plotkin SR, Temel JS, Mitchell SL, Chang Y, Barry MJ, Volandes AE. Use of video to facilitate end-of-life discussions with patients with cancer: a randomized controlled trial. Journal of clinical oncology : official journal of the American Society of Clinical Oncology. 2010;28(2):305-10. Epub 2009/12/02. doi: 10.1200/jco.2009.24.7502. PubMed PMID: 19949010; PMCID: PMC3040012.
72. Volandes AE, Paasche-Orlow MK, Mitchell SL, El-Jawahri A, Davis AD, Barry MJ, Hartshorn KL, Jackson VA, Gillick MR, Walker-Corkery ES, Chang Y, Lopez L, Kemeny M, Bulone L, Mann E, Misra S, Peachey M, Abbo ED, Eichler AF, Epstein AS, Noy A, Levin TT, Temel JS. Randomized controlled trial of a video decision support tool for cardiopulmonary resuscitation decision making in advanced cancer. Journal of clinical oncology : official journal of the American Society of Clinical Oncology. 2013;31(3):380-6. Epub 2012/12/13. doi: 10.1200/jco.2012.43.9570. PubMed PMID: 23233708; PMCID: PMC4090424.

73. Back AL, Arnold RM, Baile WF, Fryer-Edwards KA, Alexander SC, Barley GE, Gooley TA, Tulsky JA. Efficacy of communication skills training for giving bad news and discussing transitions to palliative care. Archives of internal medicine. 2007;167(5):453-60. Epub 2007/03/14. doi: 10.1001/archinte.167.5.453. PubMed PMID: 17353492.
74. Tulsky JA, Arnold RM, Alexander SC, Olsen MK, Jeffreys AS, Rodriguez KL, Skinner CS, Farrell D, Abernethy AP, Pollak KI. Enhancing communication between oncologists and patients with a computer-based training program: a randomized trial. Annals of internal medicine. 2011;155(9):593-601. Epub 2011/11/02. doi: 10.7326/0003-4819-155 9-201111010-00007. PubMed PMID: 22041948; PMCID: PMC3368370.
75. Volandes AE, Ferguson LA, Davis AD, Hull NC, Green MJ, Chang Y, Deep K, Paasche-Orlow MK. Assessing end-of-life preferences for advanced dementia in rural patients using an educational video: a randomized controlled trial. Journal of palliative medicine. 2011;14(2):169-77. Epub 2011/01/25. doi: 10.1089/jpm.2010.0299. PubMed PMID: 21254815.
76. Volandes AE, Barry MJ, Chang Y, Paasche-Orlow MK. Improving decision making at the end of life with video images. Medical decision making : an international journal of the Society for Medical Decision Making. 2010;30(1):29-34. Epub 2009/08/14. doi: 10.1177/0272989x09341587. PubMed PMID: 19675323.
77. Volandes AE, Ariza M, Abbo ED, Paasche-Orlow M. Overcoming educational barriers for advance care planning in Latinos with video images. Journal of palliative medicine. 2008;11(5):700-6. Epub 2008/07/01. doi: 10.1089/jpm.2007.0172. PubMed PMID: 18588401.
78. Volandes AE, Paasche-Orlow MK, Barry MJ, Gillick MR, Minaker KL, Chang Y, Cook EF, Abbo ED, El-Jawahri A, Mitchell SL. Video decision support tool for advance care planning in dementia: randomised controlled trial. BMJ (Clinical research ed). 2009;338:b2159. Epub 2009/05/30. doi: 10.1136/bmj.b2159. PubMed PMID: 19477893; PMCID: PMC2688013.
79. Volandes AE, Mitchell SL, Gillick MR, Chang Y, Paasche-Orlow MK. Using video images to improve the accuracy of surrogate decision-making: a randomized controlled trial. Journal of the American Medical Directors Association. 2009;10(8):575-80. Epub 2009/10/08. doi: 10.1016/j.jamda.2009.05.006. PubMed PMID: 19808156; PMCID: PMC2782701.
80. Volandes AE, Lehmann LS, Cook EF, Shaykevich S, Abbo ED, Gillick MR. Using video images of dementia in advance care planning. Archives of internal medicine. 2007;167(8):828-33. Epub 2007/04/25. doi: 10.1001/archinte.167.8.828. PubMed PMID: 17452547.
81. Epstein AS, Volandes AE, Chen LY, Gary KA, Li Y, Agre P, Levin TT, Reidy DL, Meng RD, Segal NH, Yu KH, Abou-Alfa GK, Janjigian YY, Kelsen DP, O'Reilly EM. A randomized controlled trial of a cardiopulmonary resuscitation video in advance care planning for progressive pancreas and hepatobiliary cancer patients. Journal of palliative medicine. 2013;16(6):623-31. Epub 2013/06/04. doi: 10.1089/jpm.2012.0524. PubMed PMID: 23725233.

82. Volandes AE, Levin TT, Slovin S, Carvajal RD, O'Reilly EM, Keohan ML, Theodoulou M, Dickler M, Gerecitano JF, Morris M, Epstein AS, Naka-Blackstone A, Walker-Corkery ES, Chang Y, Noy A. Augmenting advance care planning in poor prognosis cancer with a video decision aid: a preintervention-postintervention study. Cancer. 2012;118(17):4331-8. Epub 2012/01/19. doi: 10.1002/cncr.27423. PubMed PMID: 22252775; PMCID: PMC3359413.
83. Back AL, Arnold RM, Baile WF, Tulsky JA, Barley GE, Pea RD, Fryer-Edwards KA. Faculty development to change the paradigm of communication skills teaching in oncology. Journal of clinical oncology : official journal of the American Society of Clinical Oncology. 2009;27(7):1137-41. Epub 2009/01/28. doi: 10.1200/jco.2008.20.2408. PubMed PMID: 19171703; PMCID: PMC4804341.
84. Back AL, Arnold RM, Tulsky JA, Baile WF, Fryer-Edwards KA. Teaching communication skills to medical oncology fellows. Journal of clinical oncology : official journal of the American Society of Clinical Oncology. 2003;21(12):2433-6. Epub 2003/06/14. doi: 10.1200/jco.2003.09.073. PubMed PMID: 12805343.
85. Clayton JM, Adler JL, O'Callaghan A, Martin P, Hynson J, Butow PN, Laidsaar-Powell RC, Arnold RM, Tulsky JA, Back AL. Intensive communication skills teaching for specialist training in palliative medicine: development and evaluation of an experiential workshop. Journal of palliative medicine. 2012;15(5):585-91. Epub 2012/03/22. doi: 10.1089/jpm.2011.0292. PubMed PMID: 22433021.
86. Clayton JM, Butow PN, Waters A, Laidsaar-Powell RC, O'Brien A, Boyle F, Back AL, Arnold RM, Tulsky JA, Tattersall MH. Evaluation of a novel individualised communication-skills training intervention to improve doctors' confidence and skills in end-of-life communication. Palliative medicine. 2013;27(3):236-43. Epub 2012/06/20. doi: 10.1177/0269216312449683. PubMed PMID: 22711714.
87. Fryer-Edwards K, Arnold RM, Baile W, Tulsky JA, Petracca F, Back A. Reflective teaching practices: an approach to teaching communication skills in a small-group setting. Academic medicine : journal of the Association of American Medical Colleges. 2006;81(7):638-44. Epub 2006/06/27. doi: 10.1097/01.acm.0000232414.43142.45. PubMed PMID: 16799286.
88. Temel JS, Greer JA, Muzikansky A, Gallagher ER, Admane S, Jackson VA, Dahlin CM, Blinderman CD, Jacobsen J, Pirl WF, Billings JA, Lynch TJ. Early palliative care for patients with metastatic non-small-cell lung cancer. The New England journal of medicine. 2010;363(8):733-42. Epub 2010/09/08. doi: 10.1056/NEJMoa1000678. PubMed PMID: 20818875.
89. Hanson LC, Zimmerman S, Song MK, Lin FC, Rosemond C, Carey TS, Mitchell SL. Effect of the Goals of Care Intervention for Advanced Dementia: A Randomized Clinical Trial. JAMA internal medicine. 2016. Epub 2016/11/29. doi: 10.1001/jamainternmed.2016.7031. PubMed PMID: 27893884.
90. Bakitas M, Lyons KD, Hegel MT, Balan S, Brokaw FC, Seville J, Hull JG, Li Z, Tosteson TD, Byock IR, Ahles TA. Effects of a palliative care intervention on clinical outcomes in patients with advanced cancer: the Project ENABLE II randomized controlled trial. JAMA : the journal of the American Medical

Association. 2009;302(7):741-9. Epub 2009/08/20. doi: 10.1001/jama.2009.1198. PubMed PMID: 19690306; PMCID: PMC3657724.
91. Bakitas MA, Tosteson TD, Li Z, Lyons KD, Hull JG, Li Z, Dionne-Odom JN, Frost J, Dragnev KH, Hegel MT, Azuero A, Ahles TA. Early Versus Delayed Initiation of Concurrent Palliative Oncology Care: Patient Outcomes in the ENABLE III Randomized Controlled Trial. Journal of clinical oncology : official journal of the American Society of Clinical Oncology. 2015;33(13):1438-45. Epub 2015/03/25. doi: 10.1200/jco.2014.58.6362. PubMed PMID: 25800768; PMCID: PMC4404422.
92. Thorpe KE, Zwarenstein M, Oxman AD, Treweek S, Furberg CD, Altman DG, Tunis S, Bergel E, Harvey I, Magid DJ, Chalkidou K. A pragmatic-explanatory continuum indicator summary (PRECIS): a tool to help trial designers. Journal of clinical epidemiology. 2009;62(5):464-75. Epub 2009/04/08. doi: 10.1016/j.jclinepi.2008.12.011. PubMed PMID: 19348971.
93. Thorpe KE, Zwarenstein M, Oxman AD, Treweek S, Furberg CD, Altman DG, Tunis S, Bergel E, Harvey I, Magid DJ, Chalkidou K. A pragmatic-explanatory continuum indicator summary (PRECIS): a tool to help trial designers. CMAJ : Canadian Medical Association journal = journal de l'Association medicale canadienne. 2009;180(10):E47-57. Epub 2009/04/18. doi: 10.1503/cmaj.090523. PubMed PMID: 19372436; PMCID: PMC2679824.
94. Dyer N, Sorra JS, Smith SA, Cleary PD, Hays RD. Psychometric properties of the Consumer Assessment of Healthcare Providers and Systems (CAHPS(R)) Clinician and Group Adult Visit Survey. Medical care 2012;50 Suppl:S28-34 doi: 10.1097/MLR.0b013e31826cbc0d
