# Supplementary material for: Association of an Advance Care Planning Video and Communication Intervention With Documentation of Advance Care Planning Among Older Adults: A Nonrandomized Controlled Trial
Source: JAMA Netw Open. 2022 Feb 24;5(2):e220354. doi: 10.1001/jamanetworkopen.2022.0354 (PMC8874350; doi:10.1001/jamanetworkopen.2022.0354)
Supplement: Supplement 2. — eMethods. Study Organization and Committees eTable 1. Keyword Library Used for NLP Annotations eTable 2. Total Number of Video Views by Video Type, Language, and Modality eTable 3. Video View Watched Percentage eTable 4. Patient Characteristics Limited to Patients Who Appeared in All Three Periods (Sensitivity Analysis) eTable 5. NLP ACP Documentation Rates by Domain and Overall Limited to Patients Who Appeared in All Three Periods (Sensitivity Analysis) eTable 6. Rate Difference Between Intervention and Baseline Periods Using Generalized Linear Regression Models with Generalized Estimating Equations Limited to Patients Who Appeared in All Three Periods (Sensitivity Analysis) eReferences [file jamanetwopen-e220354-s002.pdf]

Supplementary Online Content

Volandes AE, Zupanc SN, Paasche-Orlow MK, et al. Association of an advance care planning video and communication intervention with documentation of advance care planning among older adults: a nonrandomized controlled trial. *JAMA Netw Open*. 2022;5(2):e220354. doi:10.1001/jamanetworkopen.2022.0354

eMethods. Study Organization and Committees

eTable 1. Keyword Library Used for NLP Annotations

eTable 2. Total Number of Video Views by Video Type, Language, and Modality

eTable 3. Video View Watched Percentage

eTable 4. Patient Characteristics Limited to Patients Who Appeared in All Three Periods (Sensitivity Analysis)

eTable 5. NLP ACP Documentation Rates by Domain and Overall Limited to Patients Who Appeared in All Three Periods (Sensitivity Analysis)

eTable 6. Rate Difference Between Intervention and Baseline Periods Using Generalized Linear Regression Models with Generalized Estimating Equations Limited to Patients Who Appeared in All Three Periods (Sensitivity Analysis)

eReferences

This supplementary material has been provided by the authors to give readers additional information about their work.

© 2022 Volandes AE et al. *JAMA Network Open.*

eMethods 1. Study Organization and Committees

Regulatory Considerations

A single Institutional Review Board (IRB) of record approached this study as a multi-center trial with Dana-Farber Cancer Institute (DFCI) as the lead site SMART IRB of record (IRB # 20-600). Northwell Health is the only participating site while Boston Medical Center, DFCI, and Massachusetts General Hospital are non-participatory sites. The "reliance agreement" established by Northwell Health's regulatory board allows for the use of DFCI's Office of Human Research Subjects (OHRS) as their main regulatory agent.

Data Use Agreements between Northwell Health and DFCI are on file and adhere to the process and procedures for the protection of human subjects and electronic health record (EHR) for their covered entities. A wavier of consent was received to cover the EHR review of individuals (unless a specific research declination is on file for an individual) included in the study and for surveying clinicians who completed the VitalTalk trainings.

This study is registered on ClinicalTrials.gov (NCT-04660422).

Inclusion and Exclusion Criteria

Clinic Eligibility: Clinics affiliated with Northwell Health were eligible to be included in the study.

Clinician, Staff Eligibility: Any staff member identified by the site-PIs who are affiliated with Northwell Health.

Patient Eligibility: To be eligible for this study, individuals must have been aged 65 or over, affiliated with the Northwell Health clinic during a study period. For each of the three periods of the study, those patients over the age of 65 who are engaged (e.g., seen in person, telehealth, etc.) with the clinic during that time were included in that time period.

- We did not include individuals who are not yet adults (infants, children, teenagers)
- We did include pregnant women
- We did not include prisoners

Risks to Subjects

The potential risks were minimal given the fact that the intervention promoted learning about advance care planning (ACP), improved communication for patients and their families regarding ACP and self-determination, and the concordance between patient's wishes and the care they receive.

The major potential risk for subjects was a loss of confidentiality. Loss of confidentiality was very unlikely because specific procedures have been implemented by the research team to prevent such disclosure and these measures were maintained during the proposed study.

Adherence

Monitoring and reporting of unforeseen adverse events to the DFCI IRB was done by all investigators. Since ACP is part of the standard of care at Northwell Health, this study presented no more than minimal risk.

Risks to confidentiality of the data collected throughout the proposed study were addressed as follows: all information in the database were indexed by subject identifier only, so that even if the database server is compromised subjects could not be identified, thus maintaining the privacy of their information. Also, assurance and confidentiality of information was made to all subjects. Data was handled with the same confidentiality accorded to patient's medical records. Specific procedures protecting subject confidentiality were as follows:

- Access to data files was secured with a password-filing system (that logs entry) and was restricted to authorized staff only.
- Necessary hard-copy records containing study data of any type were kept in locked files.

© 2022 Volandes AE et al. *JAMA Network Open.*

- Master lists linking subject information with ID number will be numbered consecutively and prepared before data collection (to ensure accurate accounting).
- These lists were kept locked, in duplicate, with access only by the PIs and the other investigators.
- All project staff signed an oath of confidentiality to ensure their understanding of the terms of confidentiality required. They will be trained in specific procedures to ensure confidentiality.
- Sign-out procedures for all access to data files was strictly enforced.
- All reports and publications will preserve the subjects' anonymity.

Video Decision Aids

Video Descriptions

The following ACP videos were made available to patients in both English and Spanish.

Video Title	Description
Choosing a health care proxy	Explores the role of a health care proxy and how a patient should choose one.
Having an ACP conversation	Discusses how to explore ACP conversations with your loved ones and clinicians. Reviews different levels of medical care and values.
What is Covid-19?	Educational video on what is Covid-19, how you can get infected, and ways to protect yourself. This video was available for adults, teens, and kids versions (in English only). The Spanish version only included the adult version.
Covid-19 vaccinations	Educational video detailing Covid-19 vaccines and side effects.

Natural Language Processing

Software Specification

We used the text annotation software, ClinicalRegex,[1] to identify ACP documentation. ClinicalRegex was developed by the Lindvall Lab at DFCI, and has been applied in multiple studies to assess process-based quality measures including ACP.[2-16] Using a pre-defined ontology, the software displays clinical notes that contain highlighted keywords or phrases associated with one or more outcomes of interest. Five research staff (Emmert, Burgess, Singh, Gromova, Alvarez Suarez) received annotation training through multiple meetings with the study investigators (Moseley, Lindvall) to discuss criteria and review coding. Human annotators then reviewed clinical notes with highlighted keywords to determine if the context was relevant to the outcome of interest. If the keywords appeared out for context, the notes were labeled for exclusion. This NLP approach allows for semi-automated chart review and reduces the complexity and time required to extract text-based information from EHRs.

Outcome Ontology

The ontology for operationalizing the primary outcome of the trial, ACP documentation, included five domains that encompass key aspects of ACP: (1) goals of care conversations; (2) limitations of life-sustaining treatment; (3) palliative care involvement; (4) hospice conversations; (5) election of a surrogate decision maker. A detailed description of the refinement and validation of the keyword library used to identify domain 1-4 in the parent trial can be found in Lindvall et al. 2021.[4]

In addition to the previous domains, (5) election of a surrogate decision maker was included in ontology for ACP documentation as defined for the ACP-COVID trial. The library for this domain was generated by requesting clinicians at multiple sites of the parent trial to list phrases where they reference surrogate decision makers. These phrases were then revised to ensure that they were specific to surrogate decision maker information as it is documented at Northwell Health. The final keyword library is shown in supplementary eTable 1.

© 2022 Volandes AE et al. *JAMA Network Open.*

Annotator Training

All research staff who participation in clinical note annotation underwent a two-component training process. The first component of the training was a meeting with a ClinicalRegex specialist (Moseley) to review software installation, use, and ACP documentation ontology.

Following this meeting, all annotators received a ‘Calibration Test’ developed by the Lindvall Lab. Consisting of mock clinical narratives (without protected health information), the calibration test was designed to closely resemble the actual clinical notes that annotators would later review. In addition to testing an annotator’s understanding and application of the keyword library, the test provided the basis for comparing rates of agreement between annotators to ensure that they were all annotating clinical notes with a high degree of accuracy.

Results of the Calibration Test were assessed across all five domains. Annotators were required to achieve an accuracy greater than 70% for each domain to earn a passing score. Among six annotators the median test accuracy was 91% [range, 88% to 94%].

To determine inter-annotator reliability, Cohen's Kappa coefficient was calculated. Cohen’s Kappa coefficient is a measure of how reliability annotators are classifying certain notes as instances of ACP document whilst considering agreement that may occur by chance. Among six annotators, the median Kappa Score was 0.78 [range, 0.71 to 0.86].

© 2022 Volandes AE et al. *JAMA Network Open.*

eTable 1. Keyword Library Used for NLP Annotations

Domain	Definition	Final
Goals of care	Conversations with patients or family members about the patient's goals, values, or priorities for treatment and outcomes. Includes statements that conversation occurred as well as listing specific goals.<br><br>OR<br><br>Advance care planning was discussed, reviewed, recommended, or completed.	goc, goals of care, goals for care, goals of treatment, goals for treatment, treatment goals, family meeting, family discussion, family discussions, patient goals, patient values, quality of life, prognostic discussions, illness understanding, serious illness conversation, serious illness discussion, acp, advance care plan, advance care planning, advanced care planning, Supportive care, comfort care, comfort approach, comfort directed care, advanced care plan/goals of care, comfort measures, end of life care, what matters most, wish
Palliative Care	Documentation that specialist palliative care was discussed, patient preferences regarding seeing palliative care clinician.	Palliative care, palliative medicine, pall care, pallcare, palcare
Hospice	Documentation that hospice was discussed, prior enrollment in hospice, patient preferences regarding hospice, or assessments the patient did not meet hospice criteria.	Hospice
Code status limitations	Conversations with patients or family members about preferences for limitations to cardiopulmonay resuscitation and intubation.	dnr, dnrdni, dni, dnr/dni, do not resuscitate, do-not-resuscitate, do not intubate, do-not-intubate, no intubation, no mechanical ventilation, no ventilation, no CPR, declines CPR, no cardiopulmonary resuscitation, chest compressions, no defibrillation, no dialysis, no NIPPV, no bipap, no endotracheal intubation, no mechanical intubation, declines dialysis, refuses dialysis, shocks, cmo, comfort measures, , comfort care, Do not resuscitate/do not intubate, DNR/DNI/DNH, DNR/I, DNAR, full code
Surrogate Decision Maker	Documentation of a surrogate decision maker.	health care agent, health care proxy, HCP, HCP agent, surrogate, decision maker, proxy, health agent, power of attorney for health care, HCPOA, health care power of attorney, health care agent, HCA
Video	Video support tool was recommended or viewed.	video, videos, decision aid, shared decision making, ACP decisions

© 2022 Volandes AE et al. *JAMA Network Open.*

eTable 2. Total Number of Video Views by Video Type, Language, and Modality

Video Group	Video Type	Language	Modality	Number of Views
Choosing a Health Care Agent	ACP	English	Remote	673
Choosing a Health Care Agent	ACP	English	In-Clinic	968
COVID for Kids and Teens	COVID	English	Remote	85
COVID Information	COVID	English	In-Clinic	1
COVID Information	COVID	English	Remote	35
COVID Vaccines	COVID	English	In-Clinic	976
COVID Vaccines	COVID	English	Remote	198
Other ACP	ACP	English	In-Clinic	2
Other ACP	ACP	English	Remote	4
The Conversation	ACP	English	Remote	113
The Conversation	ACP	English	In-Clinic	968
Choosing a Health Care Agent	ACP	Spanish	Remote	22
Choosing a Health Care Agent	ACP	Spanish	In-Clinic	416
COVID Vaccines	COVID	Spanish	Remote	4
COVID Vaccines	COVID	Spanish	In-Clinic	416
The Conversation	ACP	Spanish	Remote	5
The Conversation	ACP	Spanish	In-Clinic	416
Total Number of Views				5,302

© 2022 Volandes AE et al. *JAMA Network Open.*

eTable 3. Video View Watched Percentage

Watched Percentage	Number of Views (%)
Greater than or equal to 50%	4752 (89.6%)
Less than 50%	550 (10.4%)

© 2022 Volandes AE et al. *JAMA Network Open.*

eTable 4. Patient Characteristics Limited to Patients Who Appeared in All Three Periods (Sensitivity Analysis)

	**Pre-COVID-19 (N=7,180)**	**Wave 1 COVID-19 (N=7,180)**	**Intervention Period (N=7,180)**
Age (years) — mean (SD)	81.0 (8.2)	81.3 (8.2)	81.7 (8.2)
Female sex — no. (%)	4,590 (63.9)	4,590 (63.9)	4,590 (63.9)
Race/Ethnicity — no. (%)			
Hispanic	454 (6.3)	454 (6.3)	454 (6.3)
Non-Hispanic Asian	330 (4.6)	330 (4.6)	330 (4.6)
Non-Hispanic Black	699 (9.7)	699 (9.7)	699 (9.7)
Non-Hispanic Other	313 (4.4)	313 (4.4)	313 (4.4)
Non-Hispanic White	4,986 (69.4)	4,986 (69.4)	4,986 (69.4)
Unknown	398 (5.5)	398 (5.5)	398 (5.5)
Marital Status — no. (%)			
Married	4,077 (56.8)	4,084 (56.9)	4,071 (56.7)
Widowed	1,163 (16.2)	1,166 (16.2)	1,178 (16.4)
Divorced/Separated	598 (8.3)	596 (8.3)	588 (8.2)
Single	904 (12.6)	906 (12.6)	903 (12.6)
Other	27 (0.4)	29 (0.4)	29 (0.4)
Unknown	411 (5.7)	399 (5.6)	411 (5.7)
Number of Clinic Visits — no. (%)			
1	1,926 (26.8)	2,325 (32.4)	1,923 (26.8)
2	1,544 (21.5)	1,374 (19.1)	1,498 (20.9)
3-4	1,459 (20.3)	1,386 (19.3)	1,461 (20.3)
5+	2,251 (31.4)	2,095 (29.2)	2,298 (32.0)

© 2022 Volandes AE et al. *JAMA Network Open.*

eTable 5. NLP ACP Documentation Rates by Domain and Overall Limited to Patients Who Appeared in All Three Periods (Sensitivity Analysis)

	**Intervention (N=7,180)**	**Pre-COVID-19 (N=7,180)**	**Wave 1 COVID-19 (N=7,180)**
NLP Outcome Sensitivity Analysis — no. (%)			
Goals of Care	1,516 (21.1)	1,256 (17.5)	781 (10.9)
Palliative Care	20 (0.3)	6 (0.1)	6 (0.1)
Hospice	86 (1.2)	30 (0.4)	21 (0.3)
Limitations on Life-Sustaining Treatment	211 (2.9)	132 (1.8)	95 (1.3)
Surrogate Decision Maker	1,149 (2.9)	828 (11.5)	511 (7.1)
ACP Documentation	1,562 (21.8)	1,326 (18.5)	821 (11.4)

© 2022 Volandes AE et al. *JAMA Network Open.*

eTable 6. Rate Difference Between Intervention and Baseline Periods Using Generalized Linear Regression Models with Generalized Estimating Equations Limited to Patients Who Appeared in All Three Periods (Sensitivity Analysis)

	Intervention (N=7,180)	Pre-COVID-19 (N=7,180)			Wave 1 COVID-19 (N=7,180)		
	**n (%)**	**n (%)**	**RD (95% CI)***	**P Value***	**n (%)**	**RD (95% CI)***	**P Value***
Primary Outcome							
ACP Documentation							
Overall	1,562 (21.8)	1,326 (18.5)	3.3 (-0.6, 7.6)	0.10	821 (11.4)	10.3 (6.4, 12.8)	<0.001
Subgroups							
Non-Hispanic White	1,046 (21.0)	958 (19.2)	1.8 (-2.7, 7.0)	0.38	579 (11.6)	9.4 (5.5, 12.8)	<0.001
Minority	422 (23.5)	299 (16.7)	6.8 (2.5, 10.9)	0.002	172 (9.6)	13.9 (9.3, 17.0)	<0.001
Hispanic	94 (20.7)	68 (15.2)	5.5 (0.2, 11.5)	0.04	48 (10.5)	10.2 (4.3, 14.9)	<0.001
Non-Hispanic Asian	81 (24.5)	57 (17.2)	7.3 (1.5, 13.3)	0.01	25 (7.6)	17.0 (12.3, 22.3)	<0.001
Non-Hispanic Black	192 (27.5)	123 (17.6)	9.9 (4.1, 15.5)	<0.001	68 (9.8)	17.7 (11.6, 22.1)	<0.001
**Secondary Outcomes**							
Goals of Care	1,516 (21.1)	1,256 (17.5)	3.6 (-0.6, 7.6)	0.09	781 (10.9)	10.2 (6.0, 12.4)	<0.001
Palliative Care	20 (0.3)	6 (0.1)	0.2 (0.0, 0.4)	0.02	6 (0.1)	0.2 (0.0, 0.4)	0.01
Hospice	86 (1.2)	30 (0.4)	0.8 (0.0, 1.5)	0.04	21 (0.3)	0.9 (0.5, 1.3)	<0.001
Limitations on Life-Sustaining Treatment	211 (2.9)	132 (1.8)	1.1 (0.3, 1.8)	0.005	95 (1.3)	1.6 (0.9, 2.4)	<0.001
Surrogate Decision Maker	1,149 (16.0)	828 (11.5)	4.5 (0.7, 8.1)	0.02	511 (7.1)	8.9 (5.4, 11.7)	<0.001

© 2022 Volandes AE et al. *JAMA Network Open.*

eReferences

1. ClinicalRegex. Lindvall Lab. 2021. https://lindvalllab.dana-farber.org/clinicalregex.html
2. Johnson PC, Markovitz NH, Gray TF, et al. Association of Social Support With Overall Survival and Healthcare Utilization in Patients With Aggressive Hematologic Malignancies. *J Natl Compr Canc Netw*. Oct 15 2021:1-7. doi:10.6004/jnccn.2021.7033
3. Marziliano A, Burns E, Chauhan L, et al. Patient Factors and Hospital Outcomes Associated With Atypical Presentation in Hospitalized Older Adults With COVID-19 During the First Surge of the Pandemic. *J Gerontol A Biol Sci Med Sci*. Jul 19 2021;doi:10.1093/gerona/glab171
4. Lindvall C, Deng CY, Moseley E, et al. Natural Language Processing to Identify Advance Care Planning Documentation in a Multisite Pragmatic Clinical Trial. *J Pain Symptom Manage*. Jul 14 2021;doi:10.1016/j.jpainsymman.2021.06.025
5. Agaronnik N, Lindvall C, El-Jawahri A, He W, Iezzoni L. Use of Natural Language Processing to Assess Frequency of Functional Status Documentation for Patients Newly Diagnosed With Colorectal Cancer. *JAMA Oncol*. Oct 1 2020;6(10):1628-1630. doi:10.1001/jamaoncol.2020.2708
6. Agaronnik ND, Lindvall C, El-Jawahri A, He W, Iezzoni LI. Challenges of Developing a Natural Language Processing Method With Electronic Health Records to Identify Persons With Chronic Mobility Disability. *Arch Phys Med Rehabil*. Oct 2020;101(10):1739-1746. doi:10.1016/j.apmr.2020.04.024
7. Brizzi K, Zupanc SN, Udelsman BV, et al. Natural Language Processing to Assess Palliative Care and End-of-Life Process Measures in Patients With Breast Cancer With Leptomeningeal Disease. *Am J Hosp Palliat Care*. May 2020;37(5):371-376. doi:10.1177/1049909119885585
8. Udelsman BV, Lee KC, Lilley EJ, Chang DC, Lindvall C, Cooper Z. Variation in Serious Illness Communication among Surgical Patients Receiving Palliative Care. *J Palliat Med*. Mar 2020;23(3):411-414. doi:10.1089/jpm.2019.0268
9. Lee KC, Udelsman BV, Streid J, et al. Natural Language Processing Accurately Measures Adherence to Best Practice Guidelines for Palliative Care in Trauma. *J Pain Symptom Manage*. Feb 2020;59(2):225-232 e2. doi:10.1016/j.jpainsymman.2019.09.017
10. Udelsman BV, Lilley EJ, Qadan M, et al. Deficits in the Palliative Care Process Measures in Patients with Advanced Pancreatic Cancer Undergoing Operative and Invasive Nonoperative Palliative Procedures. *Ann Surg Oncol*. Dec 2019;26(13):4204-4212. doi:10.1245/s10434-019-07757-2
11. Poort H, Zupanc SN, Leiter RE, Wright AA, Lindvall C. Documentation of Palliative and End-of-Life Care Process Measures Among Young Adults Who Died of Cancer: A Natural Language Processing Approach. *J Adolesc Young Adult Oncol*. Feb 2020;9(1):100-104. doi:10.1089/jayao.2019.0040
12. Udelsman B, Lee K, Qadan M, et al. Management of Pneumoperitoneum: Role and Limits of Nonoperative Treatment. *Ann Surg*. Jul 1 2021;274(1):146-154. doi:10.1097/SLA.0000000000003492
13. Udelsman BV, Corey KE, Lindvall C, et al. Risk factors and prevalence of liver disease in review of 2557 routine liver biopsies performed during bariatric surgery. *Surg Obes Relat Dis*. Jun 2019;15(6):843-849. doi:10.1016/j.soard.2019.01.035
14. Lindvall C, Lilley EJ, Zupanc SN, et al. Natural Language Processing to Assess End-of-Life Quality Indicators in Cancer Patients Receiving Palliative Surgery. *J Palliat Med*. Feb 2019;22(2):183-187. doi:10.1089/jpm.2018.0326
15. Udelsman B, Chien I, Ouchi K, Brizzi K, Tulsky JA, Lindvall C. Needle in a Haystack: Natural Language Processing to Identify Serious Illness. *J Palliat Med*. Feb 2019;22(2):179-182. doi:10.1089/jpm.2018.0294
16. Lilley EJ, Lindvall C, Lillemoe KD, Tulsky JA, Wiener DC, Cooper Z. Measuring Processes of Care in Palliative Surgery: A Novel Approach Using Natural Language Processing. *Ann Surg*. May 2018;267(5):823-825. doi:10.1097/SLA.0000000000002579

© 2022 Volandes AE et al. *JAMA Network Open.*
