# Supplementary material for: Association of an Advance Care Planning Video and Communication Intervention With Documentation of Advance Care Planning Among Older Adults: A Nonrandomized Controlled Trial
Source: JAMA Netw Open. 2022 Feb 24;5(2):e220354. doi: 10.1001/jamanetworkopen.2022.0354 (PMC8874350; doi:10.1001/jamanetworkopen.2022.0354)
Supplement: Supplement 3. — Data Sharing Statement [file jamanetwopen-e220354-s003.pdf]

# Data Sharing Statement

Volandes. Association of an Advance Care Planning Video and Communication Intervention With Documentation of Advance Care Planning Among Older Adults. *JAMA Netw Open*. Published February 24, 2022. doi:10.1001/jamanetworkopen.2022.0354

**Data**
**Data available:** No

**Additional Information**
**Explanation for why data not available:** We continue to do secondary analyses.
